# Atomically Correlated Phosphorus–Sulfur Sites in Carbon Nitride for Efficient Hydrogen Peroxide Production

**DOI:** 10.1002/advs.76839

**Published:** 2026-07-27

**Authors:** Adnan Ahmad, Gbemi F. Abass, Myat Thwe Naing, Cheng Yang, Denny Gunawan, Hetaishan Huang, Qingfeng Zhai, Teng Lu, Nick Cox, Liming Dai, Rose Amal, Terry J. Frankcombe, Yun Liu

**Affiliations:** ^1^ Research School of Chemistry The Australian National University Canberra Australian Capital Territory Australia; ^2^ The ARC Centre of Excellence for Carbon Science and Innovation (COE‐CSI) Sydney New South Wales Australia; ^3^ School of Science The University of New South Wales Canberra Australian Capital Territory Australia; ^4^ School of Chemical Engineering University of New South Wales Sydney New South Wales Australia

**Keywords:** buckled 2D structure, graphitic carbon nitride, H_2_O_2_ production, heteroatom co‐doping, metal‐free photocatalysis

## Abstract

Photocatalytic oxygen reduction offers a sustainable route to hydrogen peroxide (H_2_O_2_) production; however, rapid charge‐carrier recombination and sluggish oxygen‐reduction reaction (ORR) kinetics remain the primary bottlenecks. Herein, an atomically correlated phosphorus–sulfur co‐doped graphitic carbon nitride (g‐C_3_N_4_), referred to as P‐S:gCN, is demonstrated to enable efficient H_2_O_2_ photogeneration in O_2_‐saturated water without sacrificial agents. Introducing atomically correlated P‐S sites within the heptazine framework creates an asymmetric charge distribution that suppresses trap‐mediated recombination and promotes efficient charge separation. These coupled heteroatom sites also enhance oxygen adsorption and activation on the catalytic surface, directing the reaction through the selective 2e^−^ ORR pathway to increase H_2_O_2_ generation. Under visible‐light irradiation, P‐S:gCN achieves an apparent quantum yield of 6.80% at 420 nm and a solar‐to‐chemical conversion efficiency of 0.49% in pure water. Combined experimental and theoretical evidence reveals that atomically correlated P‐S sites regulate charge transport, suppress recombination, and promote selective 2e^−^ ORR more effectively than isolated dopants. This atomically correlated dual‐site engineering opens avenues for tuning charge transport, electronic structure, and the stability of reaction intermediates in metal‐free photocatalytic systems.

## Graphical abstract

Atomically correlated phosphorus–sulphur sites are introduced into graphitic carbon nitride to regulate charge distribution, suppress localized trapping, and enhance oxygen activation. The resulting metal‐free photocatalyst selectively promotes the two‐electron oxygen reduction pathway, enabling efficient hydrogen peroxide production in pure water under visible light, without sacrificial reagents.

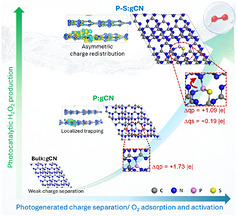

## 1 Introduction

Hydrogen peroxide (H_2_O_2_) is a green oxidant widely used in chemical synthesis, water disinfection, and energy applications [1, 2, 3]. Currently, over 95% of H_2_O_2_ is produced via the anthraquinone process, which demands extensive infrastructure and high energy input, and generates considerable chemical waste [4]. Solar‐driven photocatalytic production of H_2_O_2_ from water and oxygen has therefore emerged as a promising sustainable alternative [5, 6, 7]. However, achieving efficient H_2_O_2_ production in pure water remains challenging due to the rapid recombination of photogenerated charge carriers and limited control over reaction selectivity [8, 9].

Graphitic carbon nitride (g‐C_3_N_4_) has been extensively studied as a metal‐free photocatalyst owing to its visible‐light absorption, chemical stability, and tunable electronic structure. Its polymeric heptazine framework provides abundant nitrogen coordination sites and a conjugated π‐system suitable for photocatalytic reactions [10]. Despite these advantages, pristine g‐C_3_N_4_ suffers from limited charge‐separation efficiency and sluggish charge transfer, severely restricting its photocatalytic performance for H_2_O_2_ production [11]. To address these limitations, heteroatom doping has been widely employed to regulate the electronic structure of g‐C_3_N_4_, narrowing the band gap and generating oxophilic defect sites [12, 13]. Nonmetal dopants such as phosphorus (P) [14], sulfur (S) [15], and boron (B) [16, 17] can modify charge distribution, shift band‐edge positions, and create chemically distinct surface sites for oxygen activation [18]. However, single‐heteroatom doping often introduces highly localized electronic states that serve as charge‐trapping centers, thereby accelerating electron‐hole recombination. As a result, singly doped g‐C_3_N_4_ yields only modest overall performance gains [19].

Beyond isolated heteroatom doping, co‐doping strategies involving multiple heteroatoms have been widely explored to regulate the electronic structure of g‐C_3_N_4_ and enhance photocatalytic activity [20]. However, most reported co‐doping systems implicitly assume that two heteroatoms interact cooperatively, yet the actual spatial correlation between dopants within the carbon nitride framework is rarely verified [21, 22, 23]. Consequently, it remains unclear whether the introduced heteroatoms are electronically coupled within the same local lattice unit or coexist in spatial proximity without direct bonding. In many cases, dopants may exist independently, and the observed catalytic improvement may arise primarily from increased dopant concentration and independent electronic perturbations rather than genuine cooperative interactions [24, 25, 26, 27, 28, 29]. In this context, atomic correlation refers to heteroatom pairs that are directly coordinated or bonded locally in the crystalline structure, enabling strong electronic coupling rather than random spatial coexistence. This distinction matters because atomically correlated heteroatom pairs induce asymmetric charge redistribution within the heptazine lattice, facilitating selective two‐electron oxygen reduction (2e^−^ ORR). Therefore, understanding how local dopant‐pair correlation influences charge‐carrier dynamics and oxygen‐reduction chemistry in g‐C_3_N_4_ remains an unresolved challenge.

Here, we engineer atomically correlated P‐S sites within the g‐C_3_N_4_ framework to regulate charge‐carrier dynamics and selective oxygen reduction. Phosphorus and sulfur were selected because their differences in electronegativity and compatible p‐orbital symmetry create cooperative donor‐acceptor interactions within the g‐C_3_N_4_ lattice. Density functional theory (DFT) calculations and experimental characterizations, including X‐ray photoelectron spectroscopy (XPS) and synchrotron X‐ray absorption spectroscopy, provide evidence for atomically correlated P‐S sites embedded in the heptazine units. Unlike isolated co‐dopants, the correlated P‐S pair induces asymmetric charge redistribution, suppresses deep trap states, and enhances electronic delocalization across the framework. As a result, P‐S co‐doped g‐C_3_N_4_ (P‐S:gCN) improves charge carrier separation, favors the 2e^−^ ORR pathway, and enhances photocatalytic H_2_O_2_ production in pure water without sacrificial reagents. These findings establish atomic‐scale dopant correlation, rather than simple co‐doping, as a key design principle for regulating redox selectivity in metal‐free photocatalysts for solar‐driven H_2_O_2_ production.

## 2 Results and Discussion

To determine whether atomically correlated P‐S pairs can regulate the electronic structure of g‐C_3_N_4_ more effectively than isolated dopants, we first evaluated the energetics of representative models by DFT calculations. Dopant sites for P‐doped g‐C_3_N_4_ (P:gCN) and P‐S:gCN were identified through structural relaxation using the Perdew–Burke–Ernzerhof exchange‐correlation functional. The co‐doped configuration with P@C1 and S@N2 in direct atomic correlation is the most stable, with a formation energy of −1.04 eV. This value is substantially lower than those of all isolated P and S configurations (minimum −0.29 eV), confirming that the directly bonded P‐S motif is thermodynamically preferred over spatially separated substitution (Tables S1–S3 and Figure S3). In this configuration, P at the C1 position and S at the adjacent N2 position within the same heptazine unit are directly bonded, forming a covalent P‐S linkage embedded within the conjugated N‐P‐S‐C motif. This distinguishes the atomically correlated configuration from a scenario in which P and S independently coexist in the same material without direct chemical bonding.

Structural relaxation of the P@C1 and S@N2 configurations reveals measurable local geometric distortion relative to pristine g‐C_3_N_4_. The P─N and S─C bond lengths are significantly elongated compared to the standard C─N bond length (1.35 Å), reflecting the larger atomic radii of P (110 pm) and S (104 pm) relative to C (77 pm) and N (75 pm). This bond elongation is accompanied by measurable out‐of‐plane displacement of the S atom, as evident in the side‐view structural models (Figure S3). This geometric distortion is modest relative to the overall thermodynamic stability of the configuration, and is structurally accommodated by the intrinsic layer buckling of g‐C_3_N_4_ [30].

**FIGURE 1:**
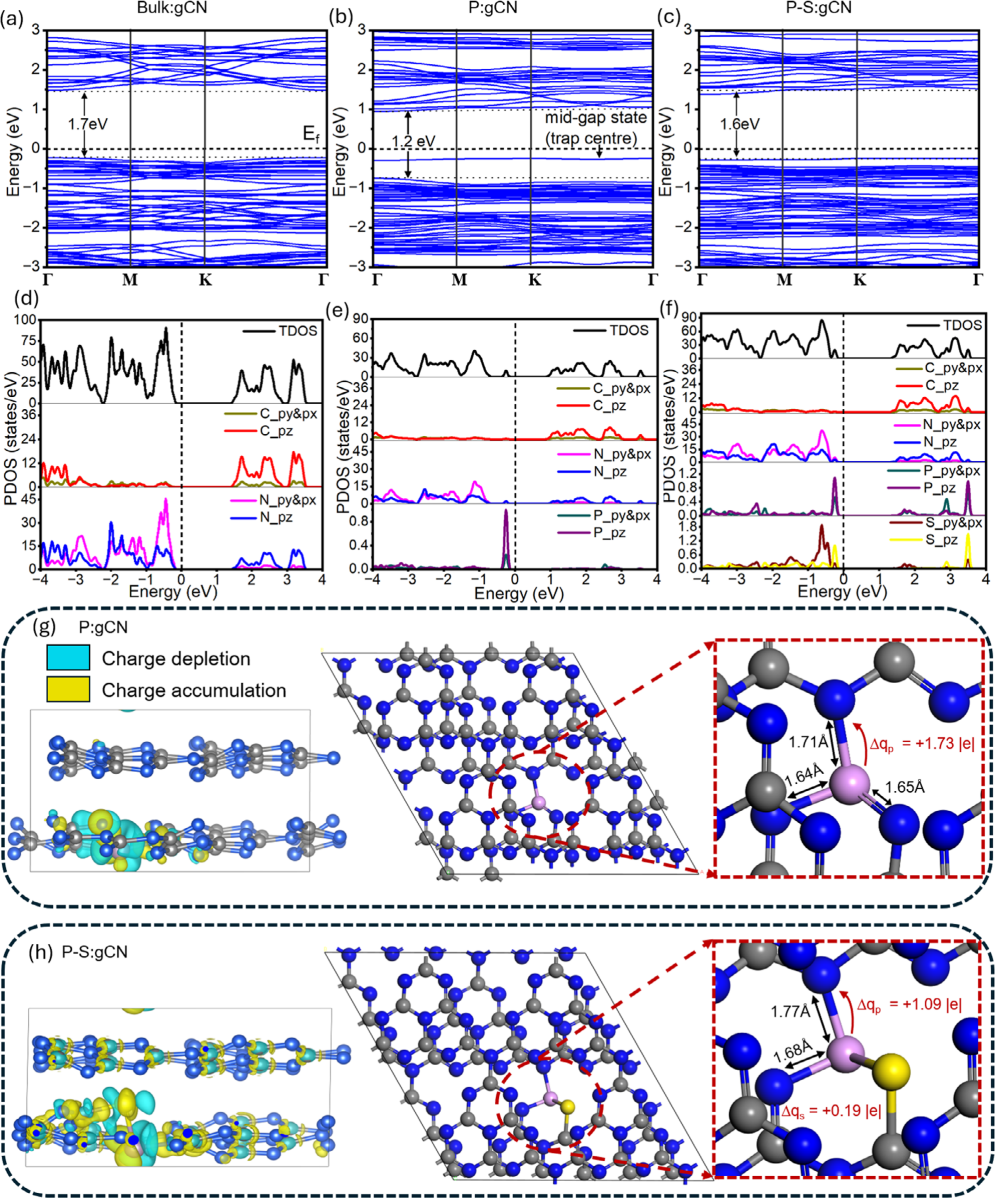
(a–c) Band structures of Bulk:gCN, P:gCN, and P‐S:gCN. (d–f) DOS of proposed models of Bulk:gCN, P:gCN, and P‐S:gCN. (g, h) CDD map (isosurface value 0.008 e/Bohr^3^) of P:gCN and P‐S:gCN bulk models, in which yellow and cyan represent electron accumulation and depletion, respectively (Light purple: P single atom. Yellow: S single atom. Blue: N atoms. Grey: C atoms).

To understand how local P‐S coordination modifies the electronic structure, the band structure and density of states (DOS) of pristine g‐C_3_N_4_ (Bulk:gCN), P:gCN, and P‐S:gCN were analyzed. As shown in Figure 1a,d, Bulk:gCN exhibits typical semiconductor features with dispersive valence band (VB) and conduction band (CB) edges, indicating delocalized π conjugation across the heptazine framework. Its VB primarily originates from N 2p states, whereas the CB mainly arises from hybridized C 2p and N 2p orbitals. Substituting the P@C1 site in the P:gCN structure narrows the bandgap; however, nearly flat defect‐derived bands emerge close to the Fermi level. The corresponding projected density of states (PDOS) shows a significant contribution from P 3p states, indicating a strong local electronic perturbation and the formation of highly localized trap‐like states (Figure 1b,e). Thus, although P doping alters the electronic structure, it also disrupts electronic delocalization and is likely to hinder efficient carrier transport. In contrast, the atomically correlated P‐S:gCN model preserves the beneficial bandgap modulation while markedly suppressing the deep localized features observed for P:gCN (Figure 1c,f). The band dispersion near the frontier states becomes more continuous, and the PDOS shows stronger hybridization among the C 2p, N 2p, P 3p, and S 3p orbitals, indicating long‐range electronic connectivity. These results indicate that direct P‐S correlation reduces trap states associated with isolated P doping and instead promotes a more delocalized electronic structure, which is better for charge transport. Charge density difference (CDD) analysis further clarifies the distinct roles of isolated and correlated dopants by revealing charge depletion and accumulation at dopant sites. In P:gCN, pronounced charge depletion occurs at the P center (Δq_P = +1.73 |e|), indicating substantial electron transfer from P to the surrounding N‐rich framework (Figure 1g). This highly localized redistribution is consistent with the defect‐state localization observed in the band structure and PDOS. By contrast, P‐S:gCN shows a more spatially distributed charge redistribution pattern throughout the lattice, indicating cooperative charge sharing between P and S (Figure 1h). The corresponding Bader charge analysis (Δq_P = +1.09 |e| and Δq_S = +0.19 |e|) indicates a more balanced redistribution of charge within the correlated‐site environment.

Such moderate local polarization reduces excessive carrier trapping and establishes a cooperative electronic environment for interfacial redox reactions. These correlated‐site electronic signatures provide the theoretical basis for the enhanced charge separation and oxygen‐reduction activity observed experimentally in P‐S:gCN.

Based on the theoretical prediction that correlated P‐S dopant pairs can modulate the electronic structure of g‐C_3_N_4_, a series of heteroatom‐doped carbon nitride samples were synthesized (Figure S1). In this approach, precursors were assembled in solution to form a homogeneous supramolecular network, which was then heated to 450°C in air for 2 h, followed by heating to 500°C under Ar for 2 h. This thermal treatment converts conjugated N‐heterocycles into heptazine units of carbon nitride. This strategy enables the controlled incorporation of heteroatoms within the heptazine framework while preserving the conjugated structure of g‐C_3_N_4_.

**FIGURE 2:**
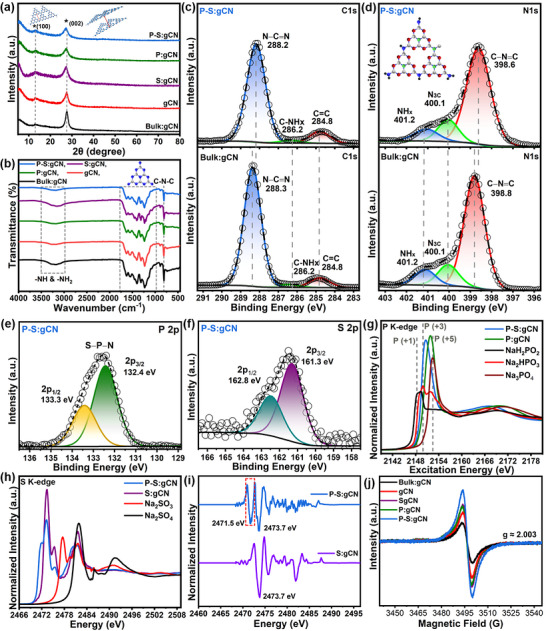
(a, b) XRD Patterns and FTIR spectra of Bulk:gCN, P:gCN, S:gCN, and P‐S:gCN. (c) High‐resolution C 1s spectra of Bulk:gCN and P‐S:gCN. (d) High‐resolution N 1s spectra of Bulk:gCN and P‐S:gCN. (e) High‐resolution P 2p spectrum of P‐S:gCN. (f) High‐resolution S 2p spectrum of P‐S:gCN. (g) P K‐edge spectra and second derivative of P‐S:gCN, P:gCN, and reference compounds (Na_3_PO_4_, Na_2_HPO_3_). (h, i) S K‐edge spectra of P‐S:gCN, S:gCN, and reference compounds (Na_2_SO_3_, Na_2_SO_4_). (j) Electron paramagnetic resonance (EPR) signals for Bulk:gCN, P:gCN, S:gCN, and P‐S:gCN.

The crystalline structure of the Bulk:gCN and doped samples was analyzed through X‐ray diffraction (XRD). Bulk:gCN displays two characteristic peaks at 2θ = 13.1° and 27.6°, indexed to the (100) and (002) reflections corresponding to in‐plane tri‐s‐triazine periodicity and interlayer stacking, respectively (Figure 2a) [31]. After heat treatment of the Bulk:gCN in Ar, the intensity of both peaks in gCN (exfoliated g‐C_3_N_4_) is substantially attenuated, indicating the in‐plane structural distortion and reduced stacking order caused by thermal etching [32]. Particularly, the (002) diffraction peak shifts to a lower angle from 27.6° (Bulk:gCN) to 27.2° (P‐S:gCN), accompanied by a noticeable intensity drop. The simultaneous attenuation and slight broadening of the (002) peaks suggest weakened long‐range stacking order due to lattice distortion induced by doping and partial delamination [14]. Furthermore, the absence of any extra XRD peaks indicates that the carbon nitride framework in P‐S:gCN remains intact with no crystalline impurity phases.

Fourier‐transform infrared (FTIR) spectrum of P‐S:gCN displays characteristic peaks that match those of Bulk:gCN (Figure 2b), indicating that the heptazine functional groups are retained after dual‐site engineering. However, FTIR alone cannot verify dopant incorporation (Figure 2b). The peaks at 809 and 889.5 cm^−1^ are attributed to out‐of‐plane triazine ring breathing and heptazine ring bending modes, respectively. The bands at 1100–1700 cm^−1^ correspond to aromatic C─N and C═N stretching modes, while those at 3000–3400 cm^−1^ are assigned to N─H stretching of terminal amino groups and O─H stretching of adsorbed water [33, 34]. Notably, the intensity ratio of the triazine breathing mode (809 cm^−1^) to the heptazine deformation mode (889.5 cm^−1^) changes from 1.95 for Bulk:gCN to 1.08 for P‐S:gCN, indicating structural perturbation upon co‐doping [35].

The deconvoluted C 1s XPS spectra of Bulk:gCN and P‐S:gCN reveal three characteristic peaks at 288.3, 286.2, and 284.8 eV, respectively (Figure 2c). These peaks are assigned to N─C═N (*sp^2^*‐hybridized C in the heptazine ring), C‐NHX (X = 1, 2, terminal amino species), and C─C (graphitic or adventitious C), respectively [36]. The N─C═N/C─C intensity ratio decreases to 8.98 in P‐S:gCN from 10.54 in Bulk:gCN, reflecting selective disruption of the conjugated N─C═N motifs upon co‐doping (Table S5) [37]. The N 1s spectra of Bulk:gCN and P‐S:gCN reveal three peaks at 398.8, 400.1, and 401.2 eV, respectively (Figure 2d). These peaks are attributed to sp^2^‐hybridized bi‐coordinated N (C═N─C), tertiary bridging N (N‐(C)_3_), and terminal amino groups (─NH_x_) within the heptazine unit, respectively [38, 39]. In addition, the high‐resolution C 1s and N 1s spectra exhibit small shifts toward lower binding energy after P‐S co‐doping (Figure 2c,d). This negative shift suggests an increase in electron density around C and N atoms within the conjugated framework. Such electronic modulation arises from heteroatom incorporation, in which P can donate electron density to the surrounding lattice, while S redistributes charge through its 3p orbitals. Similar binding‐energy shifts have been widely reported in heteroatom‐doped g‐C_3_N_4_ and are generally attributed to charge redistribution within the π‐conjugated framework. Importantly, the relatively small magnitude of the shift reflects the delocalized nature of the carbon nitride lattice, which distributes the electronic perturbation over the extended π network.

The site assignments for P and S are established by their respective high‐resolution XPS spectra. The P 2p spectrum of P‐S:gCN shows a characteristic spin‐orbit doublet at 132.4 eV (P 2p_3/2_) and 133.3 eV (P 2p_1/2_), attributable to P atoms coordinated with neighboring N and S atoms within the carbon nitride framework (Figure 2e). Compared to the P 2p doublet of P:gCN (133.5 and 134.4 eV), the peaks in P‐S:gCN shift toward lower binding energies, indicating charge redistribution around P due to coordination with S [40]. No signal characteristic of P─C bonding is detected; such features would appear 2 eV below the P‐N binding energy, confirming the absence of P‐C coordination within the detection limit [41, 42, 43]. The P 2p binding energy, therefore, establishes that P is predominantly coordinated with N within the carbon nitride framework, consistent with substitution at a C site (P@C) [44]. This spectroscopic assignment is corroborated by the observed 1.56% decrease in total C content, comparable to the atomic fraction of incorporated P (Table S6), and by DFT formation energy calculations showing that P substitution at C sites is thermodynamically preferred over N sites by 0.41–1.71 eV across all screened configurations [30]. The absence of P 2p signals at 134.5–136 eV, where surface phosphate and phosphonate groups characteristically appear, confirms that these species are not present at significant levels.

For sulfur, the S 2p XPS spectrum of P‐S:gCN shows a clear doublet at 161.3 eV (S 2p_3/2_) and 162.8 eV (S 2p_1/2_), characteristic of sulfide‐type S species in a low‐oxidation‐state bonding environment (Figure 2f) [45, 46]. The value of 161.3 eV is consistent with S in a P‐coordinated environment, where P─S bonding lowers the S 2p binding energy relative to typical C─S thioether coordination (163–164 eV) due to the lower electronegativity of P compared to C. No additional features above 166 eV are detected, ruling out oxidized S species such as sulfonate (167–168 eV) or sulfoxide (165–166 eV), and confirming that sulfur is incorporated into the carbon nitride framework rather than existing as surface oxide species [47, 48].

To probe the local coordination environment and its electronic response to P‐S co‐doping, X‐ray absorption near‐edge structure (XANES) spectroscopy was performed at the P K‐edge and S K‐edge. As shown in Figure 2g, the P K‐edge spectrum of P‐S:gCN exhibits an absorption edge position located between those of the reference compounds Na_2_HPO_3_ (P^3^^+^) and Na_3_PO_4_ (P^5^^+^). First‐derivative analysis (Figures S6 and S7, Table S7) confirms an intermediate P oxidation state in P‐S:gCN, falling between those of the P^3^^+^ and P^5^^+^ standards. Such intermediate electronic states are commonly observed for heteroatom‐doped carbon nitride frameworks, where phosphorus atoms are incorporated into the conjugated network rather than existing as fully oxidized phosphate species. This intermediate oxidation state is consistent with the P 2p binding energy from XPS, which lies between that of fully oxidized P^5^^+^ (133.5–134.0 eV) and reduced P^3^^+^ (130.8 eV) [42, 49, 50, 51]. Compared to singly doped P:gCN, the P K‐edge of P‐S:gCN is slightly red‐shifted toward lower excitation energy. This shift suggests a higher local electron density around the P atoms in the co‐doped system. The electronic enrichment can be attributed to charge redistribution arising from the coordination of P with S atoms, which modifies the local bonding environment relative to P‐N coordination alone. This trend is consistent with DFT Bader charge analysis; the P atom in P‐S:gCN carries a smaller positive charge (Δq_p_ = +1.09 |e|) than in P:gCN (Δq_p_ = +1.73 |e|), indicating reduced electron depletion around P due to the neighboring S atom. The S K‐edge XANES spectra provide further insight into the coordination environment of sulfur (Figure 2h). Both S‐doped g‐C_3_N_4_ (S:gCN) and P‐S:gCN display a dominant white line feature at 2473.5 eV, corresponding to the S 1s → σ^*^(S‐C) electronic transition, which is characteristic of sulfur incorporated into the carbon nitride framework. Especially, the P‐S:gCN spectrum exhibits an additional shoulder at approximately 2471.5 eV, which is absent in the singly doped S:gCN. This lower‐energy feature indicates S atoms directly coordinated to P, consistent with P─S bond formation rather than isolated S substitution. The corresponding second‐derivative spectra (Figure 2i) further highlight this correlated coordination. The apparent splitting of the S K‐edge peak for P‐S:gCN contrasts sharply with the single‐peak profile of S:gCN. These observations confirm the coexistence of S‐C and P‐S coordination environments in the co‐doped system and the formation of dual‐heteroatom sites within the g‐C_3_N_4_ matrix.

Room‐temperature electron paramagnetic resonance (EPR) spectra of the as‐prepared samples display an isotropic Lorentzian signal at g ≈ 2.003, attributed to unpaired electrons on sp^2^‐hybridized C atoms within the conjugated aromatic domains (Figure 2j) [52]. P‐S:gCN exhibits the most intense EPR signal, indicating a higher density of unpaired electrons at defect sites. The formation of polarized P─S bonds creates an asymmetric charge distribution within the heptazine framework, resulting in a synergistic effect of P‐S co‐doping on the electronic structure.

**FIGURE 3:**
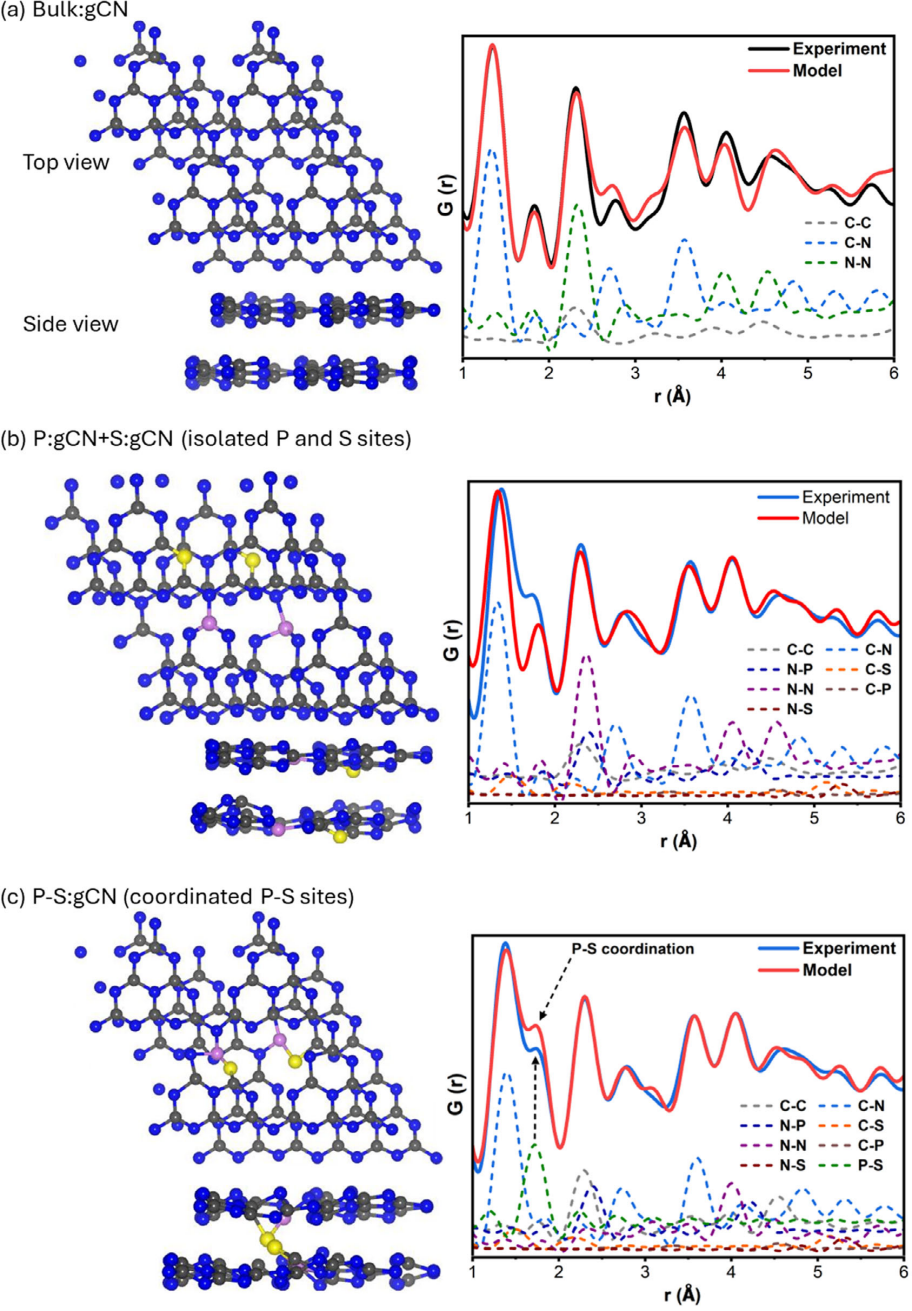
Optimized DFT structural models and PDF refinements of pristine and co‐doped gCN: (a) Bulk:gCN, (b) isolated P:gCN+S:gCN. (c) P‐S:gCN, respectively.

To resolve local atomic environments inaccessible to conventional diffraction, synchrotron X‐ray scattering combined with pair distribution function (PDF) analysis was performed. This method provides direct insights into short‐range atomic correlations and is especially effective at detecting local coordination distortions and heteroatomic linkages introduced by P and S doping. The heptazine‐based buckled structural model shows good agreement with the experimental PDF of Bulk:gCN (Figure 3a). The first peak at ∼1.35 Å corresponds to the average C─N bond distance within heptazine units. Subsequent peaks in the 2–3 Å region originate from second‐neighbor C─C and C─N correlations within the conjugated carbon nitride framework, reflecting the short‐range connectivity of heptazine layers. The refinement residual (Rw = 15.08%) indicates that the DFT‐derived structural model reproduces the experimental peak positions with good fidelity. Minor deviations in peak intensity at larger r values arise from the limited long‐range structural order of layered carbon nitride and relatively weak scattering from light elements. In contrast, the PDF profile of P‐S:gCN displays an additional feature at 1.74 Å, absent in Bulk:gCN (Figure 3b,c). The PDF peak can be assigned to P‐S local environments; only models containing directly bonded P‐S pairs at adjacent C1 and N2 sites reproduce this feature. Models with isolated P or S substitutions fail to reproduce this feature, confirming that it arises from covalent P─S bonding rather than from independent heteroatom incorporation. Corroborating this assignment, the S K‐edge XANES shoulder at 2471.5 eV, absent in singly doped S:gCN and present only in P‐S:gCN, is diagnostic of S atoms in direct coordination with P. Together, the PDF and XANES data confirm that P and S are covalently bonded within the heptazine framework, occupying adjacent substitutional sites rather than simply coexisting in spatial proximity.

Co‐substitution at adjacent C1 and N2 sites introduces a local geometric distortion, as captured directly in the PDF analysis. The first‐shell C‐N distance elongates from 1.35 to 1.39 Å with measurable peak broadening, and the N‐N pair distance increases from 2.65 to 3.08 Å. The elevated intensity between 2.3 and 3.0 Å reflects modified second‐shell interactions around P and S, accompanied by out‐of‐plane displacement at the substitution sites (Figure S3). These structural changes reflect dopant‐induced reorganization of the heptazine network and increased layer corrugation. Despite this distortion, the P@C1/S@N2 configuration retains a formation energy of −1.04 eV, substantially lower than all spatially separated dopant configurations, confirming thermodynamic preference for the directly bonded motif. To further verify the local structural configuration, several DFT‐derived models with distinct dopant distributions were refined against the experimental PDF data (Tables S8 and S9). Among these candidates, the model containing two pairs of atomically correlated P‐S sites provides the best agreement with the experimental profile, yielding the lowest refinement residual (Rw = 14.12%).

This model, therefore, represents the most realistic structural description of the synthesized P‐S:gCN. The atomic pair distances from this optimized model are summarized in Table S9 and further confirm the presence of correlated P‐S coordination within the carbon nitride lattice. With the P‐S structural configuration established, we next examined the resulting electronic and optical properties.

**FIGURE 4:**
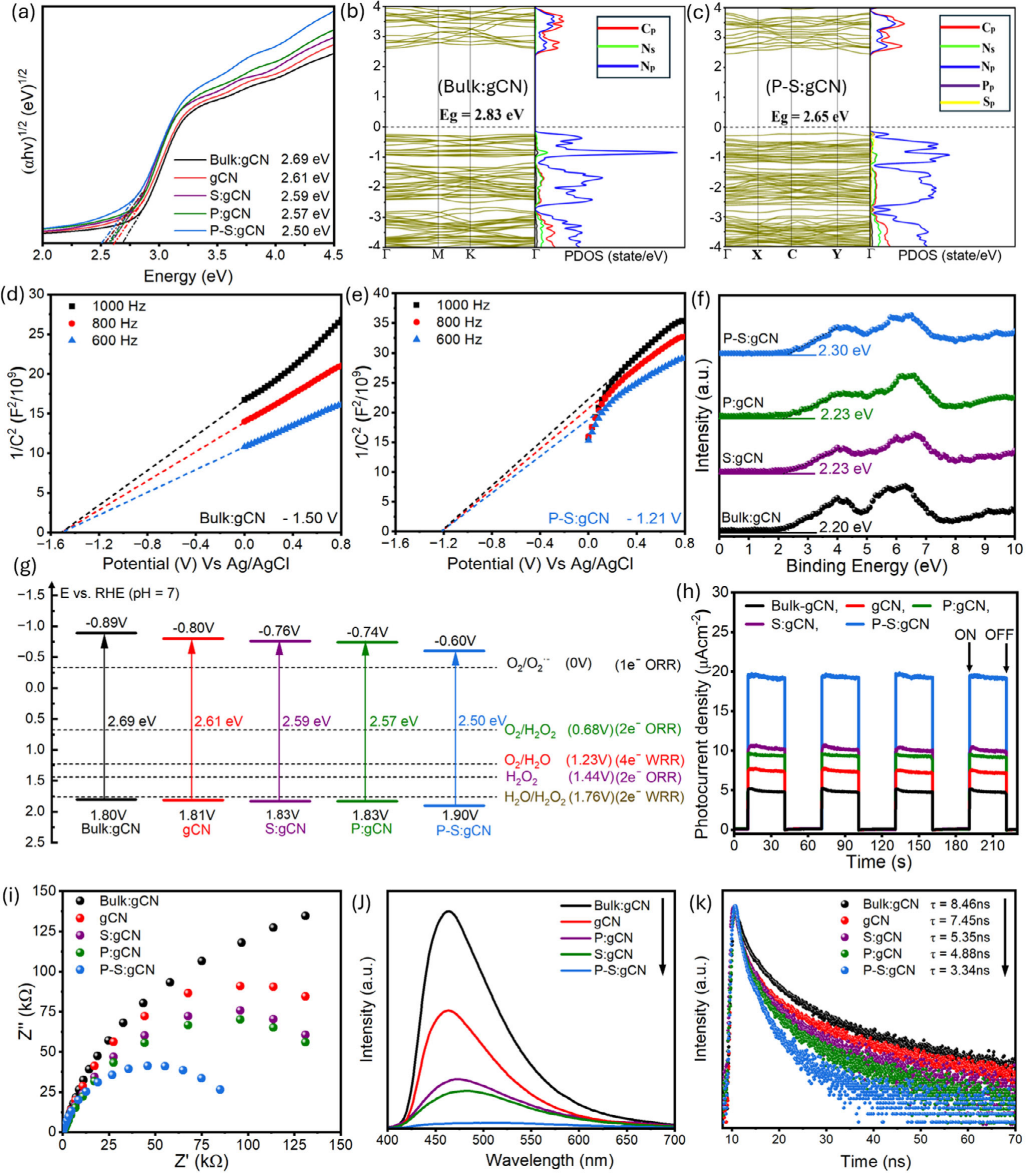
Electronic structure and band edge alignment of Bulk and co‐doped gCN systems. (a) (𝛼hν)1/2 versus hν plots. (b, c) DFT‐calculated band structure and DOS. (d, e) Mott–Schottky plots recorded at different frequencies (600–1000 Hz). (f) VB XPS spectra. (g) Band alignment diagram relative to the RHE at pH 7. (h) TPR, (i) EIS Nyquist plot, (j) Steady‐state PL spectra, and (k) TRPL of Bulk:gCN, P:gCN, S:gCN, and P‐S:gCN, respectively.

Ultraviolet–visible diffuse reflectance spectroscopy was used to evaluate the optical properties of the prepared samples (Figure S8; Figure 4a). Relative to Bulk:gCN and single‐dopant counterparts, P‐S:gCN shows a pronounced redshift and enhanced absorption across the visible region, indicating improved visible‐light utilization.

The redshift and absorption enhancement arise from dopant‐induced intraband electronic states within the π‐conjugated heptazine framework, broadening the optical response [53].

Hybrid HSE06 calculations consistently reveal a reduced bandgap for the correlated P‐S configuration, in agreement with the experimental trend. This confirms that atomic‐level dopant correlation modifies the electronic structure more effectively than isolated dopants (Figure 4b,c). To further elucidate band alignment, Mott‐Schottky and valence‐band XPS analyses were performed (Figure 4d–g). The P‐S:gCN shows a more positive flat‐band potential compared to Bulk:gCN, indicating a downward shift of the conduction band. It remains sufficiently negative relative to the reversible hydrogen electrode (RHE) to drive the thermodynamically feasible 2e^−^ ORR (E°(O_2_/H_2_O_2_) = +0.68 V vs RHE), suppressing competing multi‐electron pathways. The enhanced H_2_O_2_ production therefore stems from improved charge separation, lower interfacial charge‐transfer resistance, enhanced O_2_ activation at polarized P‐S sites, and higher 2e^−^ ORR selectivity, rather than from an increased thermodynamic driving force.

The impact of P‐S correlation on charge‐carrier dynamics was further examined using photoelectrochemical and spectroscopic techniques. Transient photocurrent response (TPR) (Figure 4h) shows that P‐S:gCN delivers the highest photocurrent density (19.5 µA cm^−2^), approximately 4‐fold that of Bulk:gCN. This enhancement demonstrates more effective separation and transport of photogenerated charge carriers. Similarly, electrochemical impedance spectroscopy (EIS) shows that P‐S:gCN exhibits the smallest semicircle radius, indicating a lower interfacial charge‐transfer resistance (Figure 4i). Steady‐state photoluminescence (PL) spectra (Figure 4j) show significantly reduced emission from P‐S:gCN relative to pristine and single‐doped samples, indicating suppressed radiative recombination. Time‐resolved photoluminescence (TRPL) analysis (Figure 4k; Table S10) further reveals a shorter average lifetime (τ_avg = 3.34 ns) for P‐S:gCN than for Bulk:gCN (8.46 ns) and gCN (7.45 ns). Although a shorter PL lifetime alone cannot distinguish accelerated charge transfer from non‐radiative recombination, the enhanced photocurrent and reduced interfacial resistance support faster carrier extraction and transport in P‐S:gCN. The combined results therefore suggest that accelerated interfacial charge transfer contributes substantially to the shortened PL lifetime, while a contribution from non‐radiative decay cannot be fully excluded.

**FIGURE 5:**
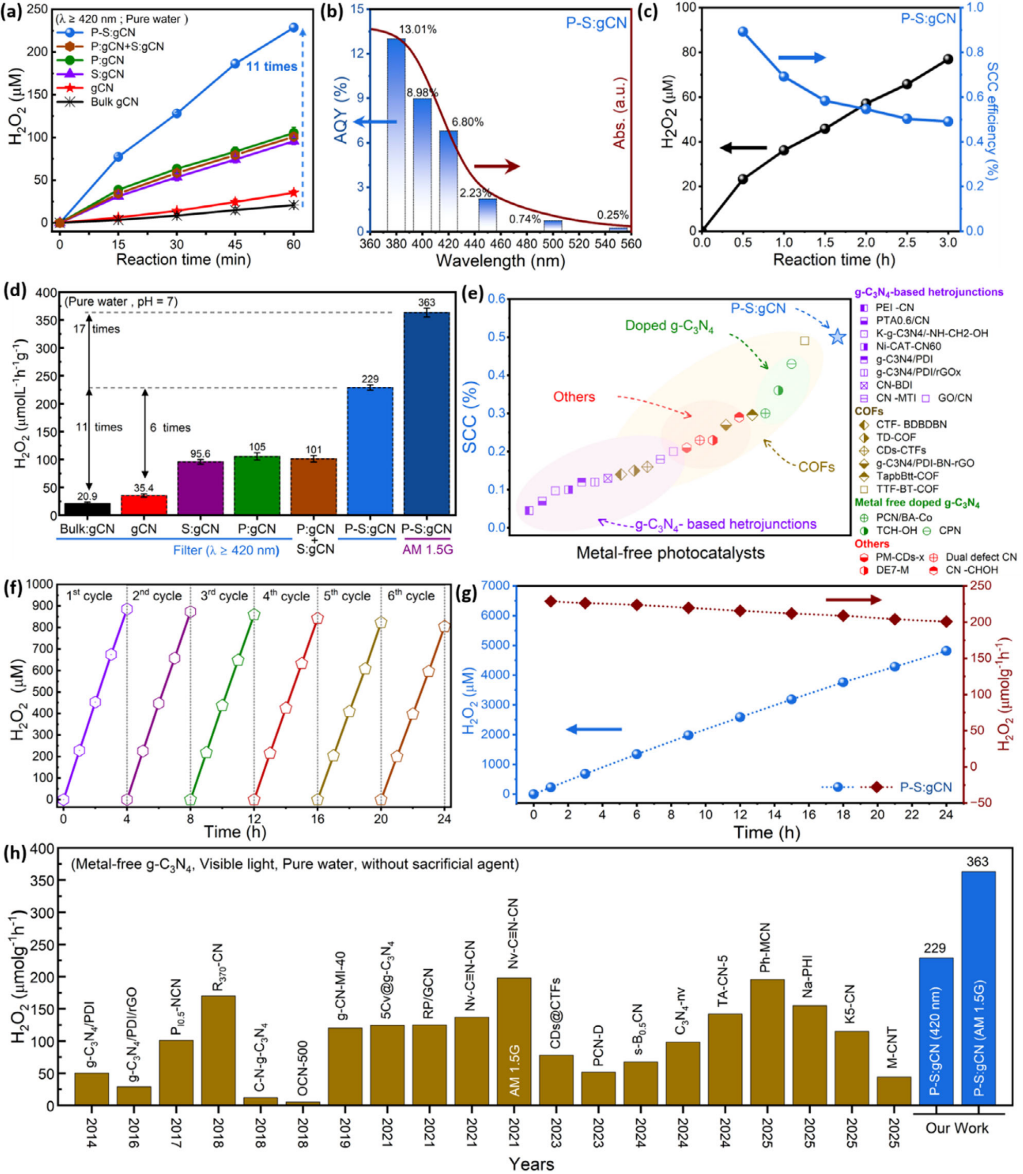
Photocatalytic H_2_O_2_ production performance of P‐S:gCN. (a) Time‐dependent H_2_O_2_ production of as‐prepared samples. (b, c) Wavelength‐dependent AQY and SCC efficiency of P‐S:gCN under simulated solar irradiation. (d) Comparison of H_2_O_2_ production rates of as‐prepared samples. (e) Benchmarking of SCC efficiency for P‐S:gCN against reported metal‐free photocatalysts in pure water [34, 56, 57, 58, 59, 60, 61, 62, 63, 64, 65, 66, 67, 68, 69, 70]. (f) Cycling stability of P‐S:gCN over six consecutive 4 h photocatalytic cycles. (g) Prolonged photocatalytic H_2_O_2_ production of P‐S:gCN. (h) Comparison of H_2_O_2_ production rates for metal‐free g‐C_3_N_4_‐based photocatalysts [60, 71, 72, 73, 74, 75, 76, 77, 78, 79, 80, 81, 82, 83, 84, 85, 86, 87].

The photocatalytic H_2_O_2_ production performance of the as‐prepared catalysts was evaluated under visible‐light irradiation (λ = 420 nm, 100 mW cm^−2^) in O_2_‐saturated pure water at neutral pH, without the use of sacrificial agents. As shown in Figure 5a, all samples exhibit a continuous increase in H_2_O_2_ concentration with irradiation time, confirming the photo‐driven nature of the reaction. Compared with Bulk:gCN (20.9 µmol g^−1^ h^−1^), gCN nanosheets show an improved rate of 35.4 µmol g^−1^ h^−1^, attributed to their larger surface area and greater active‐site accessibility for O_2_ adsorption [54]. Remarkably, P‐S:gCN delivers the highest H_2_O_2_ generation rate of 229 µmol g^−1^ h^−1^, corresponding to an 11‐fold and 6.5‐fold enhancement relative to Bulk:gCN and gCN, respectively. This activity trend is consistent with the improved carrier separation and interfacial transport indicated by the photocurrent and EIS measurements. Importantly, P‐S:gCN significantly outperforms both P:gCN (105 µmol g^−1^ h^−1^) and S:gCN (96 µmol g^−1^ h^−1^), demonstrating that the performance enhancement cannot be explained by individual heteroatom doping alone. To further distinguish between simple co‐doping and atomic‐level correlation, a physical mixture of P:gCN and S:gCN (P:gCN + S:gCN) with identical dopant ratios was prepared. This control exhibits an H_2_O_2_ production rate (101 µmol g^−1^ h^−1^) comparable to singly doped systems but substantially lower than P‐S:gCN. This result provides direct evidence that the superior activity originates from atomically correlated P‐S sites rather than the cumulative effect of independent dopants, as shown in Figure 5a–d. The influence of dopant loading was systematically investigated (Figure S11). The H_2_O_2_ production rate increases with increasing P and S content, reaching an optimal loading of 7 wt.%, beyond which a decline in activity is observed. This deterioration is attributed to structural disruption of the heptazine framework, reduced π‐conjugation, and impaired charge transport. To decouple the effect of dopant concentration from dopant interaction, P:gCN samples with equivalent total loading were also evaluated. Despite identical dopant content, P:gCN exhibits significantly lower activity than P‐S:gCN, further confirming that the enhanced performance arises from cooperative electronic interactions between correlated P‐S sites rather than dopant concentration alone (Figure S12).

To further explore the wavelength‐dependent performance of P‐S:gCN, the apparent quantum yield (AQY) was measured using a monochromatic light source. P‐S:gCN achieves an AQY of 13.01% at 380 nm and 6.80% at 420 nm (Figure 5b), consistent with its optical absorption profile. Under simulated solar irradiation, the solar‐to‐chemical conversion (SCC) efficiency reaches 0.89% in the 1st h and stabilizes at 0.49% after 3 h (Figure 5c).

Benchmarking against recently reported metal‐free g‐C_3_N_4_ photocatalysts (Figure 5e,h; Table S14) places P‐S:gCN among the top‐performing systems under sacrificial‐agent‐free conditions, confirming the effectiveness of atomic‐scale dopant correlation. Importantly, many high‐performing carbon nitride photocatalysts reported in the literature rely on hole‐scavenging sacrificial agents to achieve comparable activity, whereas P‐S:gCN operates in pure water at neutral pH without auxiliary reagents.

H_2_O_2_ accumulation is governed by a balance between formation (K_f_, µM min^−1^) and decomposition (K_d_, 10^−2^ min^−1^); both rate constants were quantified (Figure S15) [55]. P‐S:gCN delivers the highest formation rate constant (K_f_ = 4.9 µM min^−1^) and lowest decomposition rate constant (K_d_ = 0.85 × 10^−2^ min^−1^), confirming that co‐doping accelerates H_2_O_2_ generation while suppressing its catalytic breakdown.

The effect of solution pH on H_2_O_2_ production was examined to assess proton availability in the 2e^−^ ORR pathway (Figure S14). The rate increased from 229 µmol g^−1^ h^−1^ at neutral pH to 346.0 µmol g^−1^ h^−1^ at pH 3. It then decreased to 75.3 and 27.6 µmol g^−1^ h^−1^ at pH 9 and 11, respectively. This trend aligns with Figure 7f, which shows that protonation of ^*^O_2_^−^ and ^*^OOH requires available H^+^ during the 2e^−^ ORR. Enhanced H_2_O_2_ decomposition under alkaline conditions may further reduce its net accumulation. Comparable acid‐enhanced activity appears for other pure‐water photocatalysts in Table S13, indicating a broader kinetic feature rather than a material‐specific limitation. All reported performance metrics were obtained in pure, unbuffered water at native near‐neutral pH, without acid, base, buffer, or sacrificial reagents.

The operational stability of P‐S:gCN was evaluated through cycling and prolonged photocatalytic tests. Over six consecutive 4 h cycles under O_2_‐saturated conditions, P‐S:gCN retained 89.4% of its initial H_2_O_2_ production rate (202.2 µmol g^−1^ h^−1^ by the sixth cycle; Figure 5f). In a continuous 24 h run under identical irradiation conditions, P‐S:gCN preserved 88.65% of its initial production rate, with cumulative H_2_O_2_ reaching 4816 µM (Figure 5g). Post‐reaction characterization of the recovered catalyst confirms the structural and chemical integrity of the framework. The gradual rate attenuation is attributed to progressive H_2_O_2_ accumulation and mild surface passivation at millimolar product concentrations, rather than irreversible degradation of the P‐S sites. Post‐reaction XRD and FTIR spectra match those of the as‐synthesized catalyst, and all heptazine‐related C─N and C═N vibrational modes remain intact after the 24 h reaction (Figure S16a,b). High‐resolution P 2p and S 2p XPS spectra show the P 2p_3/2_ and S 2p_3/2_ binding energies unchanged at 132.4 and 161.3 eV, respectively, with no phosphate (134.5 eV) or sulfate (168 eV) features detected, confirming that the P‐S coordination sites do not undergo oxidative decomposition. XPS quantification revealed only marginal decreases in the P and S contents after 24 h, from 3.50 to 3.48 wt.% and from 3.53 to 3.49 wt.%, respectively, while the total oxygen content remained unchanged (Table S12). Elemental mapping by energy‐dispersive X‐ray spectroscopy confirms that P and S remain uniformly distributed across the nanosheets, with no evidence of dopant segregation (Figure S16e).

**FIGURE 6:**
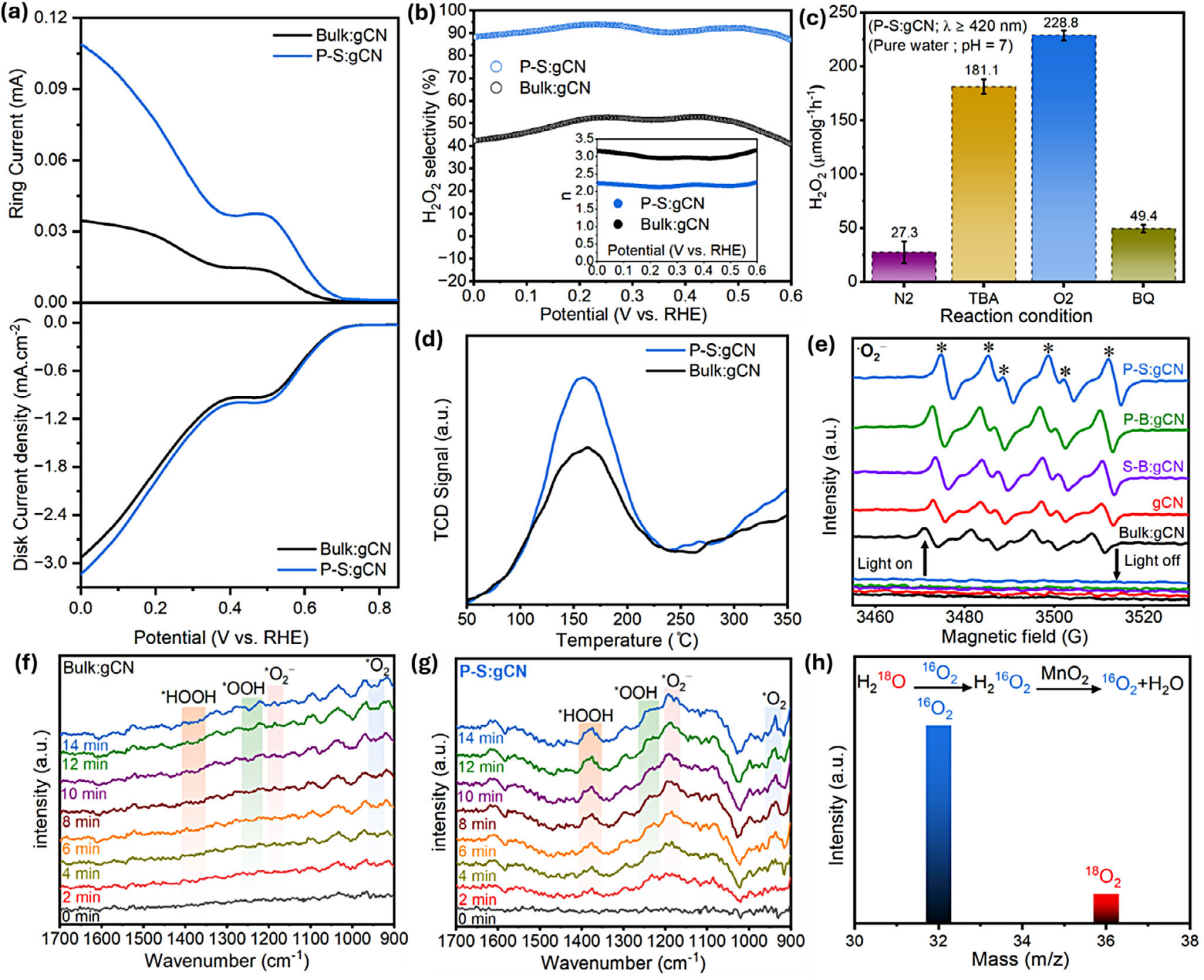
(a) RRDE polarization curves over Bulk:gCN and P‐S:gCN in O_2_‐saturated 0.1 M KOH at 1600 rpm with ring current (upper part) and disk current (bottom part). (b) H_2_O_2_ selectivity as a function of the applied potential. The inset shows the calculated average number of transferred electrons (n). (c) Photocatalytic H_2_O_2_ production in the presence of different scavengers. (d) TPD‐O_2_ of Bulk:gCN and P‐S:gCN (e) EPR signals of ·O_2_^−^ of the samples in the presence of DMPO. (e, f), respectively. (f, g) In‐situ FTIR spectroscopy of Bulk:gCN and P‐S:gCN under light illumination, revealing enhanced formation of surface intermediates including ^*^OOH and ^*^HOOH over P‐S:gCN. (h) Isotope‐labeling mass spectrometric analysis conducted in H_2_^1^8O under a ^1^ ^6^O_2_ atmosphere.

Efficient H_2_O_2_ production requires high selectivity toward the 2e^−^ ORR pathway, while suppressing competing side reactions [88]. Rotating disk electrode and rotating ring‐disk electrode (RRDE) measurements resolved the 2e^−^ and 4e^−^ ORR pathways at the electrochemical level for Bulk:gCN and P‐S:gCN. As shown in Figure 6a, both catalysts produced increasing disk and ring currents as the applied potential decreased from 0.8 V versus RHE. This confirms progressive O_2_ reduction accompanied by H_2_O_2_ formation. The disk‐current responses of the two catalysts were comparable. However, P‐S:gCN generated a substantially higher ring current than Bulk:gCN across the potential range tested. This increased ring current indicates that a larger fraction of reduced O_2_ was released as H_2_O_2_, rather than being further reduced to H_2_O. P‐S:gCN maintained an electron‐transfer number (n) close to 2 across the applied potential range, whereas Bulk:gCN exhibited a substantially higher n (> 3.00). This higher electron transfer indicates ongoing competition from deeper oxygen reduction on the pristine framework. In line with these observations, P‐S:gCN achieved an H_2_O_2_ selectivity of 92% at 0.5 V vs. RHE, compared to only 50% for Bulk:gCN (Figure 6b). Collectively, these results demonstrate that the correlated P‐S configuration promotes selective O_2_ reduction via the 2e^−^ ORR pathway while suppressing further reduction of oxygen‐derived intermediates to H_2_O.

To elucidate the role of the 2e^−^ ORR pathway in H_2_O_2_ formation, reaction‐atmosphere and scavenger experiments were conducted (Figure 6c). P‐S:gCN exhibited a marked decrease in H_2_O_2_ production under nitrogen (N_2_) compared to oxygen (O_2_), confirming that molecular O_2_ is essential for the predominant reaction pathway. The residual H_2_O_2_ detected under N_2_ is attributed to O_2_ generated in situ by photogenerated holes via the water oxidation reaction (WOR). Tert‐butanol (TBA), a hydroxyl radical scavenger, had minimal impact on H_2_O_2_ production, suggesting that hydroxyl radicals (•OH) do not significantly contribute to the main pathway. In contrast, benzoquinone (BQ), a superoxide radical (•O_2_^−^) quencher, substantially suppressed H_2_O_2_ production, thereby identifying •O_2_^−^ as a critical intermediate in the 2e^−^ ORR pathway.

Temperature‐programmed desorption (TPD) measurements revealed a significantly stronger and broader O_2_ desorption peak at 170°C for P‐S:gCN compared to Bulk:gCN, indicating substantially higher O_2_ surface uptake on the correlated framework (Figure 6d). This increased O_2_ uptake aligns with enhanced O_2_ activation, and a greater density of reactive oxygen intermediates on P‐S:gCN. The formation of •O_2_^−^ (O_2_ + e^−^ → •O_2_^−^) was further verified by 5,5‐dimethyl‐1‐pyrroline N‐oxide (DMPO) spin‐trapping EPR spectroscopy, which confirmed the involvement of superoxide species in the photocatalytic reaction. P‐S:gCN exhibited a pronounced •O_2_^−^ signal compared to dark conditions, indicating efficient electron transfer to adsorbed O_2_ and subsequent formation of •O_2_^−^ radicals (Figure 6e).

Time‐resolved in‐situ synchrotron‐radiated FTIR spectra provided direct evidence for proton‐coupled steps following superoxide formation (Figure 6g). Upon illumination, vibrational bands corresponding to •O_2_^−^, •OOH (hydroperoxyl), and HOOH (adsorbed H_2_O_2_) emerged more rapidly and with greater intensity on P‐S:gCN than on Bulk:gCN. The concurrent detection of an O─O stretching band at 892 cm^−1^ during the photocatalytic reaction indicates a two‐step, single‐electron pathway from O_2_ to H_2_O_2_, rather than a single, concerted two‐electron transfer. Preserving the integrity of the O─O The bond throughout the reaction sequence is essential for selectivity; cleavage at any intermediate stage would redirect the reaction toward H_2_O rather than H_2_O_2_.

Isotopic labeling experiments using ^1^ ^8^O confirmed the origin of the oxygen atoms in the produced H_2_O_2_. The generated H_2_O_2_ was detected by gas chromatography‐mass spectrometry following its conversion to O_2_ with MnO_2_. Photocatalytic synthesis of H_2_O_2_ over P‐S:gCN was performed in a ^1^ ^6^O_2_‐saturated H_2_^1^ ^8^O solution (Figure 6h). The predominant ^1^ ^6^O_2_ signal, resulting from decomposition of the photogenerated H_2_O_2_, indicated that the oxygen atoms in H_2_O_2_ originate primarily from molecular O_2_ rather than from water.

Together, the RRDE, scavenger, O_2_‐TPD, EPR, in‐situ FTIR, and isotope‐labeling measurements demonstrate that the P‐S correlation increases O_2_ adsorption, accelerates O_2_ activation, and promotes sequential formation of •O_2_^−^, •OOH, and •HOOH, favoring selective 2e^−^ ORR over the competing one‐electron and four‐electron pathways.

The reaction pathway can therefore be expressed as:

(1) $$*+O_{2}\to *O_{2}$$

(2) $$*O_{2}+e^{-}\to \;*{O_{2}}^{-}$$

(3) $$*{O_{2}}^{-}+H^{+}\to *\mathrm{OOH}$$

(4) $$*\mathrm{OOH}+H^{+}+e^{-}\to *\mathrm{HOOH}$$

(5) $$*\mathrm{HOOH}\;\to \;H_{2}O_{2}+*$$

(6) $$O_{2}+2H^{+}+2e^{-}\to H_{2}O_{2}$$

(7) $$2H_{2}O+4h^{+}\to O_{2\;}+4H^{+}$$

In the absence of a sacrificial hole scavenger, sustained H_2_O_2_ production in pure water requires that photogenerated holes be consumed via WOR at the same rate as photogenerated electrons are consumed via ORR. WOR also supplies the protons consumed in ORR (Equations 3 and 4), coupling the two half‐reactions both kinetically and stoichiometrically. To assess whether this coupling holds quantitatively, the oxygen balance was monitored over 24 h by measuring the net change in O_2_ within a sealed reactor, along with the amount of H_2_O_2_ produced.

**FIGURE 7:**
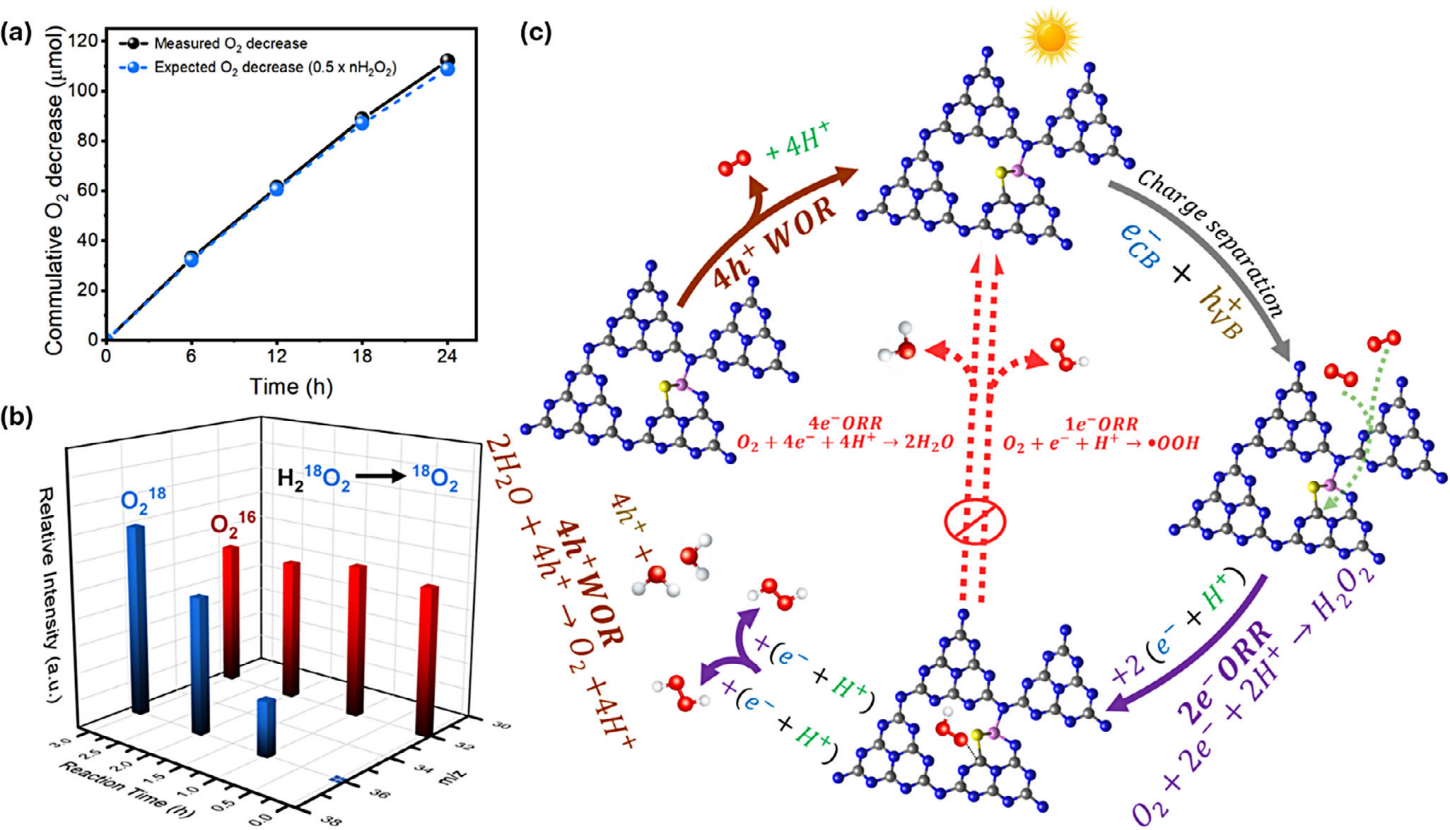
Mechanistic verification of coupled four‐electron water oxidation and 2e^−^ ORR over P‐S:gCN. (a) Time‐dependent decrease in O_2_ compared with the theoretical O_2_ consumption. (b) Isotope‐labeling analysis performed in H_2_^1^ ^8^O under a ^1^ ^6^O_2_ atmosphere. (c) Proposed photocatalytic pathway involving WOR to produce O_2_, followed by ORR to H_2_O_2_.

According to the 2e^−^ ORR pathway (Equation 6), production of one mole of H_2_O_2_ consumes one mole of O_2_ and two electrons. Charge neutrality, therefore, requires the simultaneous consumption of two photogenerated holes. In the four‐hole WOR pathway (Equation 7), four holes oxidize two moles of H_2_O to release one mole of O_2_, so the two holes required to balance the ORR generate only 0.5 mol of O_2_ via WOR. The net O_2_ consumed in the overall reaction, therefore, equals half the amount consumed by the ORR alone. The predicted O_2_ decrease was compared with the measured decrease at several time intervals (Figure 7a). The two datasets matched closely throughout the 24 h period. This agreement is captured by the stoichiometric closure, the ratio of measured to predicted O_2_ consumption, which reached 102.6%, 101.5%, 102.1%, and 103.2% at 6, 12, 18, and 24 h, respectively (Figure S18 and Table S15). These results confirm that the oxygen inventory remained quantitatively balanced during extended irradiation. The marginal excess above 100% likely reflects oxygen loss during sampling and analysis.

To further substantiate this finding, an independent product‐balance measurement quantified O_2_ evolution and H_2_O_2_ production separately, isolating the O_2_ generated by WOR from the O_2_ consumed by the 2e^−^ ORR. After 24 h, the reaction yielded approximately 119 µmol O_2_ and 240 µmol H_2_O_2_, giving an O_2_/H_2_O_2_ ratio of 0.49, consistent with the theoretical value of 0.5 (Figure S19). This ratio remained close to 0.5 throughout the reaction, with no systematic drift over time. This indicates that framework oxidation and other uncontrolled side reactions do not significantly account for the O_2_ loss observed in the closed‐system measurement (Figure 7).

To verify that the generated O_2_ originated from WOR rather than dissolved background O_2_, isotope‐resolved gas analysis was conducted using H_2_^1^ ^8^O. The gas‐phase ^1^ ^8^O_2_ signal increased steadily throughout irradiation, confirming that the detected O_2_ derives from water rather than residual dissolved gas (Figure 7b). DMPO‐•OH spin‐trapping experiments further rule out the competing 4e^−^ ORR pathway. P‐S:gCN showed no DMPO‐•OH signal under either dark or illuminated conditions (Figure 7e; Figure S20), indicating that illumination does not generate detectable free •OH, the key intermediate of the 4e^−^ pathway.

Combined with the water‐derived ^1^ ^8^O_2_ formation, these results support a complete four‐hole WOR pathway to O_2_ on P‐S:gCN, rather than partial radical formation capable of decomposing H_2_O_2_ or degrading the framework. This is further supported by post‐reaction XRD, FTIR, and XPS analyses of the catalyst after 24 h, which showed no new phosphate or sulfate features and no increase in total oxygen content (Figure S16 and Table S12).

The mechanism illustrated in Figure 7c is proposed based on these results. Visible‐light excitation generates electron‐hole pairs within P‐S:gCN, which are directed into two coupled redox half‐reactions. Photogenerated electrons migrate to O_2_ adsorption sites, where sequential electron and proton transfer converts adsorbed O_2_ to ^*^O_2_^−^, ^*^OOH, and ^*^HOOH before H_2_O_2_ is released (Equations (1), (2), (3), (4), (5)). Simultaneously, photogenerated holes oxidize water via the 4h^+^ pathway (Equation 7), providing both O_2_ and protons necessary to sustain the ORR. Efficient charge separation, atomically correlated sites that promote end‐on O_2_ adsorption, and suppression of freely diffusing •OH radicals contribute to the observed selectivity and durability. Collectively, these features enable direct H_2_O_2_ production from water and molecular oxygen over P‐S:gCN.

## 3 Conclusion

This work addresses rapid electron‐hole recombination in metal‐free gCN photocatalysts through atomic‐level heteroatom pairing. We introduce a rational co‐doping strategy that creates atomically correlated P‐S pairs within the heptazine framework, thereby overcoming the limitations associated with single‐dopant P or S sites. DFT calculations indicate that P‐S coordination is thermodynamically preferred over separate substitutional sites, a conclusion supported by the PDF structural refinement. The cooperative N‐P‐S‐C bonding motif reconfigures the local electronic environment, inducing asymmetric charge redistribution and suppressing trap‐mediated recombination that is prevalent in single‐dopant counterparts. As a result, P‐S:gCN achieves an 11‐fold enhancement in H_2_O_2_ production relative to Bulk:gCN, an AQY of 6.80% at 420 nm, and an SCC efficiency of 0.49% in pure water without sacrificial agents. Mechanistically, the correlated P‐S sites regulate oxygen activation and stabilize key intermediates, thereby promoting selective 2e^−^ ORR while suppressing competing pathways. This work establishes atomic‐scale dopant correlation as a governing principle in photocatalytic performance. It provides a general design principle for developing efficient metal‐free photocatalysts for solar‐to‐chemical energy conversion.

## Author Contributions

**Adnan Ahmad**: conceptualization, investigation, methodology, validation, visualization, formal analysis, data curation, Writing – review and editing, Writing – original draft, software. **Gbemi F. Abass**: software, investigation, data curation. **Liming Dai**: investigation, writing – review and editing, resources, validation. **Qingfeng Zhai**: investigation. **Teng Lu**: investigation, writing – review and editing. **Rose Amal**: visualization, validation, writing – review and editing, resources. **Myat Thwe Naing**: investigation. **Yun Liu**: conceptualization, writing – review and editing, supervision, resources, project administration, funding acquisition. **Hetaishan Huang**: investigation. **Cheng Yang**: investigation, formal analysis, software. **Terry J. Frankcombe**: formal analysis, supervision, investigation, writing – review and editing. **Denny Gunawan**: investigation. **Nick Cox**: investigation, writing – review and editing.

## Conflicts of Interest

The authors declare no conflicts of interest.

## Supporting information




**Supporting File**: advs76839‐sup‐0001‐SuppMat.docx.

## Data Availability

The data that support the findings of this study are available from the corresponding author upon reasonable request.
