# Supplementary material for: Atomically Correlated Phosphorus–Sulfur Sites in Carbon Nitride for Efficient Hydrogen Peroxide Production
Source: Adv Sci (Weinh). 2026 Jul 27:e76839. Online ahead of print. doi: 10.1002/advs.76839 (PMC13403726; doi:10.1002/advs.76839)
Supplement: Supplementary file 1 — Supporting File: advs76839‐sup‐0001‐SuppMat.docx. [file ADVS-9999-e76839-s001.docx]

**Supporting Information**

**Atomically Correlated Phosphorus-Sulfur Sites in Carbon Nitride for Efficient Photocatalytic Hydrogen Peroxide Production**

*Adnan Ahmad^1,2†^, Gbemi F. Abass^2,3†^, Myat Thwe Naing^1,2^, Cheng Yang^1,2^, Denny Gunawan^2,4^, Hetaishan Huang^2,4^, Qingfeng Zhai^2,4^, Teng Lu^1^, Nick Cox^1,2^, Liming Dai^2,4^, Rose Amal^2,4^, Terry J. Frankcombe^2,3*^, and Yun Liu^1,2*^*

^1^Research School of Chemistry, The Australian National University, Canberra, ACT 2601, Australia

^2^ The ARC Centre of Excellence for Carbon Science and Innovation (COE-CSI), Australia

^3^School of Science, The University of New South Wales, Canberra, ACT 2612, Australia

^4^School of Chemical Engineering, University of New South Wales, Sydney, NSW, 2052 Australia

*Email:* [*yun.liu@anu.edu.au*](mailto:yun.liu@anu.edu.au) , *t.frankcombe@unsw.edu.au*

† These authors contribute equally to this work.

**Supporting Methods**

**Supporting Figures 1-20**

**Supporting Tables 1-15**

**Supporting References 1-24**

# Experimental section

## Synthesis of samples

Urea (CH₄N₂O), phosphoric acid (H₃PO₄), ammonium thiocyanate (NH₄SCN), silver nitrate (AgNO₃), lanthanum oxide (La₂O₃), tert-butyl alcohol (TBA), benzoquinone (BQ), 5,5-dimethyl-1-pyrroline N-oxide (DMPO), Nafion solution (5 wt%), isopropyl alcohol, sodium sulfate (Na₂SO₄), potassium hydroxide (KOH), hydrogen peroxide standard solution (30 w/w%), deionized water, oxygen gas (O₂, 99.999%), nitrogen gas (N₂, 99.999%), and argon gas (Ar, 99.999%). All chemicals were of analytical grade and used as received without further purification.

## Synthesis of pristine graphitic carbon nitride and exfoliated carbon nitride nanosheets

Briefly, 10.0 g of urea was placed into alumina crucibles, covered with a lid, and heated in a muffle furnace. The temperature was then increased from room temperature to 450 °C at 5 °C/min and held for 2 hours in air. After calcination, the resulting powder was cooled to room temperature, collected, and ground into a fine powder. This powder was washed five times with distilled water to remove impurities, then dried in a vacuum oven at 80 °C for 12 hours to produce pristine carbon nitride (g-C_3_N_4_), labeled as Bulk:gCN.

To prepare exfoliated carbon nitride nanosheets, 2 g of the as-prepared Bulk:gCN was calcined in a tube furnace at 500 °C with a ramp rate of 2 °C/min for 2 hours under an argon (Ar) atmosphere. After cooling the quartz boat, the sample was collected, ground into a fine powder, and washed five times with distilled water. The powder was then dried under vacuum at 80 °C for 12 hours. Subsequently, 100 mg of the dried powder was ultrasonically exfoliated in 100 ml of pure water for 8 hours, and the suspension was collected by centrifugation. The exfoliated powder was labeled as gCN.

## Synthesis of phosphorus and sulfur doped gCN (P-S:gCN)

In this synthesis, 5.0 g (83.25 mmol) of urea, 5 g (65.68 mmol) of thiourea, and varying amounts of phosphoric acid 0.025 g (0.255 mmol), 0.050 g (0.510 mmol), 0.075 g, (0.765 mmol), 0.10 g (1.02 mmol), 0.125 g (1.276 mmol), and 0.150 g (1.53 mmol), depending on the P loading, were dissolved in 50 mL of deionized water. Similarly, different amounts of ammonium thiocyanate, 0.019 g (0.255 mmol), 0.039 g (0.510 mmol), 0.058 g (0.765 mmol), 0.078 g (1.020 mmol), 0.097 g (1.276 mmol), and 0.117 g (1.531 mmol) were added to the solution to control the S loading into the samples. The solvent was removed under vacuum at 60 °C for 12 hours to obtain a white supramolecular powder. The mixed powder was transferred to a covered crucible and heated to 450 °C at 5 °C/min in a muffle furnace under air for 2 hours. The calcined powder was collected and ground after cooling to room temperature. To exfoliate the prepared powder, it was further calcined in a tube furnace at 500 °C with a ramp rate of 2 °C/min for 2 hours under an Ar atmosphere. After cooling to room temperature, the powder was collected, washed five times with distilled water, and dried in a vacuum oven at 80 °C for 12 hours. Then, 100 mg of the dried powder was ultrasonically exfoliated in 100 mL of purified water for 8 hours, and the suspension was collected by centrifugation. The exfoliated powder was dried under vacuum at 80 °C for 12 hours. The resulting samples were labeled as x_P-S:gCN, where x represents the molar concentration, based on the loading amount. The optimal sample, in terms of photocatalytic performance, with 1.02 mmol of both phosphoric acid and ammonium thiocyanate, is designated as P-S:gCN.

## Control of atomically correlated P-S sites in P-S:gCN

To promote atomically correlated P-S local sites within the g-C₃N₄ framework, the synthesis was designed as a precursor-guided supramolecular assembly followed by thermal condensation (Figure S1). Phosphoric acid and ammonium thiocyanate were introduced in equimolar amounts to achieve comparable P and S incorporation during polymerization. During the low-temperature assembly stage (60 °C, vacuum drying, 12 h), urea/thiourea and the P/S-containing precursor species interact through hydrogen bonding and electrostatic attraction. These interactions promote local proximity between the two dopant precursors before formation of the covalent heptazine framework. Subsequent condensation at 450-500 °C converts the assembled precursor into atomically correlated P-S sites.

DFT calculations further indicate that the local proximity of P and S is energetically favoured. Among all examined bulk configurations, P@C1/S@N2, in which P and S occupy adjacent substitutional sites within the same heptazine unit, exhibits the lowest formation energy of -1.04 eV. This value is 0.65 eV lower energy than that of the most stable spatially separated configuration, 1P@C4/S@N5, with a formation energy of -0.39 eV (Table S3).

Spectroscopic and structural characterization of the final material support this assignment. High-resolution P 2p XPS shows a lower binding-energy shift for P-S:gCN relative to singly doped P, indicating a modified local electronic environment around P (Figure 2e). S K-edge XAS reveals an additional low-energy shoulder at approximately 2471.5 eV, absent in S, consistent with a P-associated S environment (Figure 2h). PDF analysis identifies a correlation feature at 1.74 Å that is reproduced only by structural models containing atomically correlated P-S sites (Figure 3). EDS elemental mapping confirms uniform nanoscale distribution of P and S across the nanosheets, with no evidence of dopant-rich segregated regions (Figure S16). Collectively, the precursor-directed synthesis, relative formation energies, XPS, XAS, and PDF results support the preferential formation of atomically correlated P-S configurations in P-S:gCN.


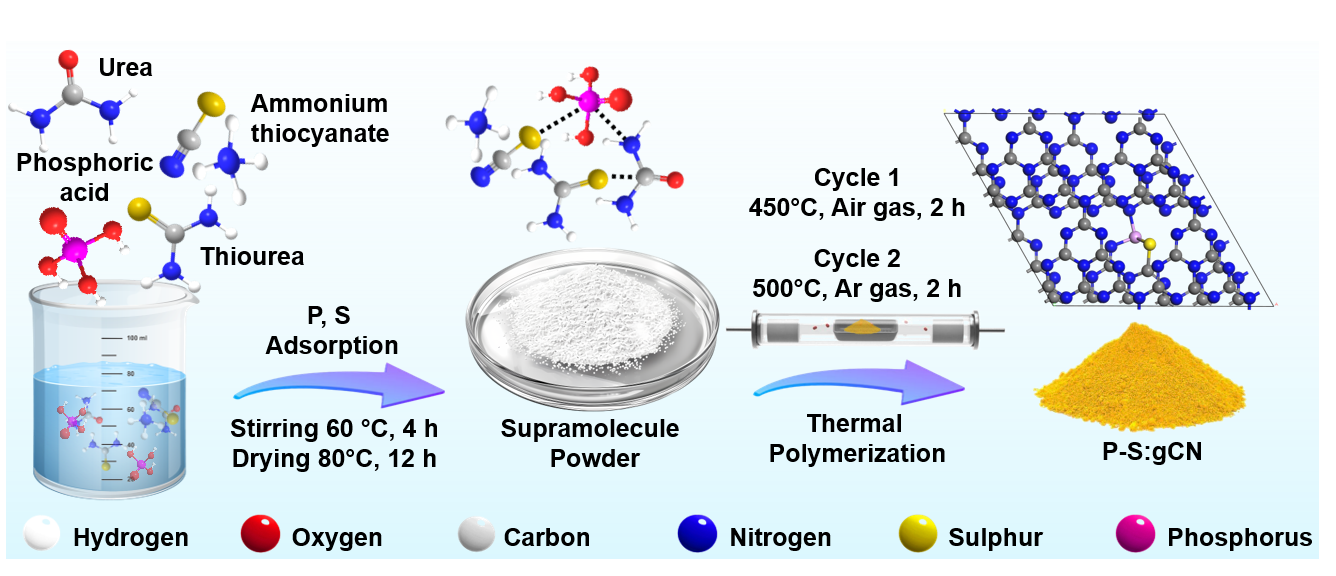


Figure S1. Schematic illustration of the preparation process for P-S:gCN.

# Characterization

The X-ray diffraction (XRD) spectra of the as-prepared samples were recorded on a Malvern Panalytical Empyrean XRD instrument using λ = 1.54 Å. The diffracted intensity of Cu Kα radiation at 40 kV and 40 mA was recorded over a 2θ range from 5° to 80°. Fourier transform infrared (FTIR) spectra, in the range of 4000-500 cm⁻¹, were collected with a PerkinElmer Spectrum Two FT-IR Spectrometer equipped with an attenuated total reflectance accessory with a KRS-5 crystal. X-ray photoelectron spectroscopy (XPS) measurements were performed on a Thermo Scientific ESCALAB 250 Xi using Al Kα excitation, with the C1S peak calibrated at 284.8 eV. The surface morphology and microstructure of the samples were characterized using scanning electron microscopy (SEM, Zeiss Ultra Plus FESEM) and transmission electron microscopy (TEM, JEOL 2100F). Energy-dispersive X-ray spectroscopy (EDS) was used for element mapping on a JEOL 2100F TEM. Ultraviolet-Visible diffuse reflectance spectroscopy (UV-Vis DRS) was conducted on a V-770 double-beam spectrophotometer (JASCO Corp., Tokyo, Japan) equipped with a deuterium/halogen light source, a single Czerny-Turner monochromator with dual gratings, and dual detectors (PMT for 190-900 nm; Peltier-cooled PbS for 800-2700 nm).

The P and S K-edge X-ray absorption spectroscopy (XAS) measurements were conducted at the Medium Energy XAS 2 (MEX2) beamline at the Australian Synchrotron, ANSTO. The synchrotron operated at 3 GeV with a beam current of 200 mA. Powder samples were mounted on carbon tape. XAS measurements were performed in drain-current and fluorescence modes under high vacuum (1 × 10^-5^ mbar).

Photoluminescence (PL) and time-resolved photoluminescence (TRPL) spectra were measured using an FS5 spectrofluorometer (Edinburgh Instruments, Livingston, UK) equipped with a 150 W ozone-free xenon arc lamp, dual Czerny–Turner monochromators (0–30 nm continuously adjustable band-pass), and a photon-counting R928P PMT detector. For the PL spectra, an excitation wavelength of 365 nm was used, while the TRPL spectra were collected with an excitation wavelength of 375 nm.

The N₂ adsorption-desorption isotherms and pore-size distributions of the prepared photocatalyst were measured with a Micromeritics 3-Flex instrument under pure N₂ gas flow. Before measurement, the samples were degassed at 150 °C for 12 hours to remove residual gas and water.

Temperature-programmed desorption (TPD) of O_2_ measurements was conducted using a Micromeritics Autochem II. A 50 mg sample of the prepared catalyst was loaded into the reaction tube and analyzed using the following procedure: First, the sample was pre-treated at 150°C at a rate of 10°C/min under a helium (He) flow for 1 hour, then cooled to 50°C under He gas. The reaction tube was saturated with a 1% O_2_/He gas mixture for 2 hours. The gas was switched back to He to remove weakly adsorbed oxygen, and the sample was held for 1 hour until the baseline stabilized. All gases were supplied at a constant flow rate of 20 ml/min. Finally, the reaction tube temperature was increased to 400°C at a heating rate of 10°C/min for O_2_ desorption, monitored by a thermal conductivity detector (TCD). The temperature was maintained at 400°C for 30 minutes to complete the desorption process.

In-situ synchrotron-radiated Fourier-transform infrared (SR-FTIR) spectroscopy was conducted on the Infrared Microspectroscopy (IRM) beamline at the Australian Synchrotron using a customized reactor. The photocatalyst was deposited onto copper tape, and a thin layer of air-purged deionized water was applied on top. A 5 mm UV light-emitting diode (LED, λ = 395 nm) was used as the light source. Measurements were performed in reflection mode with vertically incident infrared light, using 256 scans at a resolution of 4 cm⁻¹.

X-ray total scattering measurements were performed at beamline ID31 at the European Synchrotron Radiation Facility (ESRF) with an incident X-ray energy of 75.051 keV (λ = 0.16520 Å).

## Photocatalytic hydrogen peroxide (H_2_O_2_) generation reaction

30 mg of photocatalyst was loaded into a photochemical cell containing 30 mL of deionized water at pH 7, and the mixture was sonicated for 30 minutes to disperse the catalyst uniformly. The photochemical cell was sealed and purged with O₂ for 30 minutes in the dark. After reaching O₂ saturation, the flow rate was set to 18 L/h for the duration of the reaction. The photocatalytic reaction was performed under a 300 W Xe lamp (Perfect Light) equipped with a 420 nm cutoff filter (λ ≥ 420 nm), with continuous magnetic stirring and O₂ flow. The average light intensity was maintained at 100 mW/cm², and chilled fluid was circulated through the photochemical cell to keep the reaction temperature at 25 °C. Every 15 minutes, 2 mL of the reaction mixture was extracted from the cell and filtered to remove the photocatalyst. The amount of H₂O₂ generated during the photochemical reaction was measured using the iodometric test, as described in the next section. The impact of H⁺ concentration on photocatalytic H₂O₂ production was investigated by adjusting the pH level with perchloric acid (HClO₄) and potassium hydroxide (KOH). To explore the effect of different light irradiation conditions on H₂O₂ photogeneration, the AM 1.5G filter was used on the lamp instead of the 420 nm cutoff filter.

## Quantification of H₂O₂

The iodometric method was employed to determine the H_2_O_2_ content produced during the photochemical reaction. Briefly, 0.5 mL of potassium hydrogen phthalate (C_8_H_5_KO_4_) solution (0.1 mol L^-1^) and 0.5 mL of potassium iodide (KI) solution (0.4 mol L^-1^) were mixed with 1.5 mL of the extracted solution and kept for 60 minutes. The concentration of H_2_O_2_ in the mixture was measured by absorbance at 350 nm using a UV-Vis spectrophotometer (BRand). Essentially, $I^{-}$from the KI reacted with H_2_O_2_, converting into $I^{-3}$in acidic conditions, according to the following reaction ($H_{2}O_{2}+3I^{-}+2H^{+}\to I^{-3}+2H_{2}O$), which shows a strong absorption peak at 350 nm. The total amount of H_2_O_2_ produced was determined using a calibration curve for the standard solution (Supplementary Figure S7a).

## Determination of apparent quantum yield (AQY)

The AQY efficiency of the P-S:gCN was measured by performing a photochemical reaction using 50 mg of the photocatalyst in 50 mL of pure water with a pH of 7. The experiment was conducted under irradiation from a 300 W Xe lamp (PLS-SXE300E, PerfectLight) with cutoff filters at 400, 420, 450, 500, and 550 nm. Light intensities for each filter were measured with an optical power meter (Thorlabs), and the irradiation area was maintained at 1.70 cm². The photoreaction lasted 1 hour to determine the AQY, which was calculated using the following equation.

$$AQY=\frac{2\times H_{2}O_{2} content (mol)}{the number of incident photons (mol)}\times100\%$$

## Determination of solar to chemical conversion (SCC)

The SCC efficiency for the P-S:gCN was measured under light irradiation using a 300 W Xe lamp (PLS-SXE300E, PerfectLight) with an AM 1.5G filter to simulate sunlight in pure water. The irradiated area was set to 1.70 cm², and the light intensity was adjusted to 100 mW/cm² by controlling the lamp power, as monitored by an optical power meter (Thorlabs). The SCC efficiency was calculated using the following equation.

$$S=\frac{\left[ {\Delta G}_{H_{2}O_{2}} \right]\times\left[ n_{H_{2}O_{2}} \right]}{I\times S\times T}\times100\%$$

Where ${\Delta G}_{H_{2}O_{2}}$represents the Gibbs free energy for H_2_O_2_ generated (117 KJ mol^-1^), $n_{H_{2}O_{2}}$ is the molar amount of H_2_O_2_ formed, $I,S,\mathrm{and} T$ are the light intensity of simulated sunlight (100 mW.cm^-2^), irradiated area, and illuminated time (s), respectively.

## Cycling and long-term photocatalytic stability tests

The photocatalytic stability of P-S:gCN was evaluated under the same reaction conditions used for H₂O₂ production in pure water. For the cycling test, each photocatalytic cycle was conducted for 4 h under visible-light irradiation (λ ≥ 420 nm) in an O₂-saturated aqueous suspension. After each cycle, the catalyst was recovered, washed with deionized water, and re-dispersed in fresh pure water for the next cycle. The H₂O₂ concentration was quantified after each cycle using the iodometric method.

For the long-term stability test, P-S was continuously irradiated for 24 h under identical O₂-saturated pure-water conditions. After the 24 h reaction, the catalyst was recovered, washed with deionized water, dried, and characterized by XRD, FTIR, high-resolution P 2p and S 2p XPS, XPS elemental analysis, and EDS elemental mapping to evaluate post-reaction structural and chemical stability.

## Electrochemical measurement of oxygen reduction reaction (ORR)

The number of transferred electrons and H_2_O_2_ selectivity during the ORR reaction were measured using a rotating ring-disk electrode (RRDE). A three-electrode system was used in the electrochemical measurements, with Ag/AgCl as the reference electrode, RRDE as the working electrode, and Pt/C as the counter electrode. The electrolyte was a 0.1 M KOH solution saturated with O_2_. The RRDE was rotated at 1600 rpm, and a potential range of 0 to 1.0 V vs RHE was applied.

To prepare the RRDE working electrode, 4 mg of catalyst was dispersed in 400 µL of pure water, 600 µL of isopropanol, and 10 µL of Nafion solution, then sonicated for 1 hour. A 10 µL drop of this ink was applied to the RRDE electrode and dried at room temperature. The following equation is used to calculate the number of transferred electrons in ORR:

$$n=\frac{4I_{disk}}{I_{disk}+ I_{ring}/N}$$

The H_2_O_2_ selectivity of the prepared samples is determined according to the equation below.

$$H_{2}O_{2} selectivity \left( \% \right)=2\times\frac{I_{ring}/N}{I_{disk}+ I_{ring}/N}\times100\%$$

Where $I_{ring}$ represents the ring current, $I_{disk}$ is the disk current, and N is the collection efficiency, which is 0.35.

## Photoelectrochemical reduction measurement

The electrochemical impedance spectroscopy (EIS), Mott-Schottky plots, and transient photocurrent response (TPR) were measured using a three-electrode system with an AUTOLAB PGSTAT204 electrochemical workstation. A thin catalyst film was coated on an indium tin oxide (ITO) substrate, serving as the working electrode. In contrast, an Ag/AgCl electrode functioned as the reference, and a platinum plate served as the counter electrode. A 0.2 M Na₂SO₄ solution acted as the electrolyte. The photoelectrochemical cell was purged with high-purity argon gas (99.999%) for 30 minutes before and during the experiment. All photoelectrochemical measurements were conducted at room temperature (approximately 25°C) for all samples.

## H_2_O_2_ decomposition test

To determine H_2_O_2_ decomposition by the photocatalyst, 30 mg of photocatalyst were dispersed in a 1 mM H_2_O_2_ aqueous solution by ultrasonication for 30 min. The reaction parameters were identical to the photocatalytic H_2_O_2_ generation reaction. However, N_2_ gas was purged before and during the decomposition reaction instead of O_2_.

## Photocatalytic oxygen production

The photocatalytic oxygen production was tested under visible light irradiation (≥420 nm) in a closed photochemical cell connected to a gas chromatography (GC) system (Agilent 8890). 30 mg of photocatalysts were dispersed in an aqueous solution using ultrasonication for 30 minutes. After dispersion, 20 mM of silver nitrate (AgNO_3_), as an electron acceptor, was added to the reaction solution. To remove all residual gas, argon was purged through the sealed reactor for 30 minutes. The O_2_ gas produced during the photocatalytic reaction was monitored with an online GC system (5 Å molecular sieve column, Ar carrier gas), and the total amount was determined from a calibration curve prepared with standard O_2_ gas.

## Experiments with different sacrificial agents

The reactive species trapping experiment was conducted using the sacrificial agents tert-butyl alcohol (TBA) and benzoquinone (BQ) to examine how the scavengers affect H_2_O_2_ production. 1 mM of both BQ and TBA was added to the solution during their respective reactions, while keeping all other reaction conditions the same as those in the photocatalytic H_2_O_2_ production experiment. To study the impact of N_2_ on the photogeneration of H_2_O_2_, N_2_ gas was purged into the reactor for 30 minutes instead of O_2_, and a steady N_2_ flow (18 LPH) was maintained throughout the photocatalytic reaction.

## Spin-trapping (electron paramagnetic resonance) EPR measurement

By employing the in-situ EPR measurement, the spin-trapping experiment was carried out with visible light exposure (λ ≥ 420 nm), using 5,5-dimethyl-1-pyrroline N-oxide (DMPO) as a spin trap, to detect the peroxide radical (•O_2_^-^). Briefly, 2 mg of photocatalyst (2 g/L) was ultrasonically dispersed in 1 mL of a methanol solution for 30 minutes. After uniform dispersion, 100 μL of solution was loaded into the vial, followed by the addition of 10 L of DMPO aqueous solution (50 mg/mL), which was then mixed evenly. Then, the dispersion solution containing DMPO was loaded into the Suprasil low-temperature aqueous cell, followed by in situ EPR measurement under light irradiation.

## Isotope labelling Test

The isotope-labelling experiments were conducted following a reported procedure. [1]. Briefly, 10 mg of catalyst was dispersed in 1 mL of H₂¹⁸O (97 atom% ¹⁸O) in a sealed quartz reactor equipped with gas-tight fittings. The reactor was evacuated to remove residual air, after which O₂ was bubbled through the suspension for 30 min. The suspension was then stirred in the dark for an additional 30 min to establish adsorption–desorption equilibrium. Before irradiation, the headspace gas was analysed by gas chromatography mass spectrometry (GC-MS) as a control. After 6 h of irradiation, the gaseous products in the reactor headspace were analysed using a GC-MS system (GCMS-QP2020 NX, Shimadzu). The photogenerated H₂O₂ was subsequently decomposed using MnO₂ under an Ar atmosphere, and GC-MS analysed the resulting O₂. The isotopic composition of the evolved O₂ was used to determine whether the oxygen atoms in H₂O₂ originated from H₂¹⁸O through water oxidation or from molecular O₂ through the oxygen-reduction pathway.

## Stoichiometric balance measurement

The stoichiometric relationship between O₂ consumption and H₂O₂ production was evaluated in sealed quartz photoreactors. Each reactor had a total internal volume of 100 mL and contained 50 mg of P-S:gCN dispersed in 50 mL of deionized water, resulting in a headspace volume of 50 mL. The initial solution pH was 7, and no sacrificial reagent was added. Before irradiation, the catalyst suspension was purged with O₂ for 30 min to establish an O₂-saturated reaction environment. The reactor was then sealed and irradiated using a 300 W xenon lamp equipped with a 420 nm cutoff filter. The reaction temperature was maintained at 25 °C throughout the experiment. A 0.5 mL gas sample was withdrawn from the reactor headspace and analysed by gas chromatography to determine the remaining O₂ content. The H₂O₂ concentration in the liquid phase was quantified using the same analytical procedure applied in the photocatalytic activity measurements. The amount of O₂ present in the reactor headspace at each time point was calculated using:

n(O₂)ₜ = y(O₂,ₜ)PVₕ / RT

where y(O₂,ₜ) is the measured mole fraction of O₂, P is the reactor pressure, Vₕ is the headspace volume (50 mL), R is the ideal gas constant, and T is the absolute temperature.

The experimentally observed decrease in O₂ was determined from:

Δn(O₂)observed = n(O₂)₀ − n(O₂)ₜ

where n(O₂)₀ and n(O₂)ₜ are the initial and remaining amounts of O₂, respectively.

The amount of H₂O₂ produced was calculated from:

n(H₂O₂)ₜ = C(H₂O₂,ₜ)Vₗ

where C(H₂O₂,ₜ) is the measured H₂O₂ concentration and Vₗ is the liquid volume.

According to the overall reaction:

2H₂O + O₂ → 2H₂O₂

One mole of O₂ is consumed for every two moles of H₂O₂ formed. The theoretical O₂ consumption was therefore calculated as:

Δn(O₂)theoretical = 0.5n(H₂O₂)ₜ

The stoichiometric closure was calculated using:

Stoichiometric closure (%) = [Δn(O₂) observed / 0.5n(H₂O₂)ₜ] × 100

A closure of 100% indicates exact agreement between the experimentally measured O₂ decrease and the amount theoretically required for the detected H₂O₂ production. Values slightly above or below 100% reflect the combined uncertainty associated with gas sampling, GC quantification, reactor volume, and H₂O₂ analysis.

## Computation work

All periodic first-principles calculations in this work were conducted within the framework of plane-wave density functional theory (DFT) using the Vienna Ab initio Simulation Package (VASP). The electron–ion interactions were described using the projector-augmented wave (PAW) method, with valence electrons treated explicitly. Exchange-correlation effects were approximated using the generalized gradient approximation (GGA) in the form of the Perdew–Burke-Ernzerhof (PBE) functional. To accurately capture long-range dispersion interactions, which are essential for describing interlayer coupling in bulk g-C₃N₄, van der Waals corrections were incorporated using the DFT-D3 scheme with Becke-Johnson damping.

To assess the robustness of energetic trends and electronic structure features, selected configurations were further examined using the r²SCAN meta-GGA functional. At the same time, single-point energy calculations were performed using the screened hybrid Heyd–Scuseria-Ernzerhof (HSE06) functional. A plane-wave kinetic energy cutoff of 500 eV was employed throughout, and electronic self-consistency was achieved with a total-energy convergence criterion of 10⁻⁶ eV.

Structural models were constructed using 2 × 2 supercells of the heptazine-based g-C₃N₄ framework, explicitly including the intrinsic pore motif. For two-dimensional models, a vacuum separation of approximately 20 Å was introduced along the out-of-plane direction to eliminate spurious periodic interactions. Brillouin-zone integrations were carried out using Γ-centered Monkhorst-Pack k-point meshes of 3 × 3 × 1 for slab geometries and 3 × 3 × 7 for bulk structures. These k-point grids were validated through systematic convergence tests to ensure the accuracy of the total energy.

To explore low-energy non-planar configurations and capture experimentally relevant structural corrugation, controlled out-of-plane atomic displacements (0.5 Å) were introduced for selected atoms within the heptazine units to break ideal planar symmetry intentionally. All atomic structures were fully relaxed until the residual Hellmann–Feynman forces on each atom were below 0.02 eV Å⁻¹.

The thermodynamic stability of substitutional P and S dopants in both monolayer and bulk g-C₃N₄ was evaluated using spin-polarized DFT calculations, accounting for possible defect-induced localized magnetic moments. The substitutional defect formation energy ($E_{form}^{sub})$ was defined as:

$$E_{form}^{sub}= E_{doped}-E_{undoped}-\mu_{dopant}+\mu_{C/N}$$

where $E_{\mathrm{doped}}$and $E_{\mathrm{undoped}}$denote the total energies of the doped and pristine supercells, respectively. $\mu_{C/N}$is the chemical potential of the dopant species, which corresponds to the chemical potential of the substituted host atom (C or N). The chemical potentials were referenced to their most thermodynamically stable elemental phases: black phosphorus for P, α-sulfur for S, graphite for C, and molecular nitrogen (N₂) calculated in a large periodic supercell for N. All reference phase calculations were independently converged with respect to k-point sampling to ensure that numerical uncertainties in the formation energies were negligible relative to the energetic differences discussed in this study.

# Identification of the dopant sites through DFT modeling

The thermodynamically most stable dopant configuration of P-gCN and P-S:gCN is determined using density functional theory (DFT) calculations. The dopant sites for P:gCN and P-S:gCN were identified through systematic DFT screening of all inequivalent substitution sites within the heptazine unit, including corner carbon (C1), bay carbons (C4 and C6), and bridging nitrogen atoms (N2, N5, and N_bridge), using the PBE exchange-correlation functional with DFT-D3 dispersion correction (Figure S2). This screening approach follows the methodology established in our recent structural study of g-C₃N₄, which systematically evaluated all C (C1-C6) and N (N1-N7) substitution sites and demonstrated that P substitution at C sites is thermodynamically preferred over N sites by 0.41-1.71 eV across all tested configurations [2]. Building on this foundation, the present work extends the screening to P-S co-doping configurations. Among all tested combinations, the P@C1/S@N2 configuration, featuring an atomically correlated P-S pair within the same heptazine unit, is the most thermodynamically stable, with a formation energy of -0.69 eV in the monolayer and -1.04 eV in the bulk model (Figure S3). These values are substantially lower than those of spatially separated P and S configurations, where the minimum formation energy is -0.29 eV (Tables S1-S3, Figure S3). This energetic preference indicates that local P-S correlation is thermodynamically favored over isolated heteroatom substitution, and that the dopant site selection is grounded in systematic energetic screening rather than assumption. Additionally, the bulk configuration for pristine and doped g-C_3_N_4_ consistently shows lower formation energy than the monolayer structure, indicating that the bulk buckled model for g-C_3_N_4_ provides a more accurate structural representation.

**
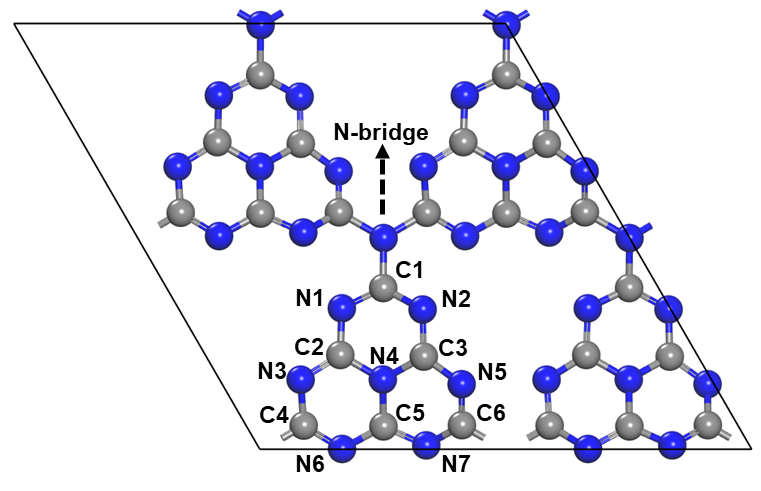
**

Figure S2. Difference substitution sites of C and N into the CN matrix, especially defined with Inequivalent C and N substitution sites considered in the g-C₃N₄ model.”

Table S1. Phosphorus and sulfur co-doping configurations and formation energies of P-S:gCN in a single sheet DFT models

| **Sr.** | **Co-dopants sites** | **E_f_ (eV)** | **Structural Model** | |
| --- | --- | --- | --- | --- |
| **One P and one S substitution sites** | | | **Top View** | **Side View** |
| 01 | 1P@C1 & S@N2 | -0.69 | 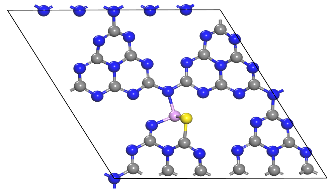 | 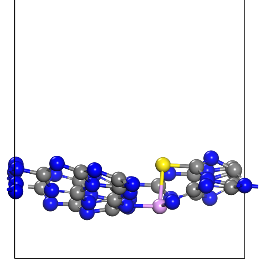 |
| 02 | 1P@C1 & S@N5 | -0.06 | 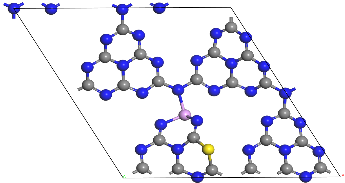 | 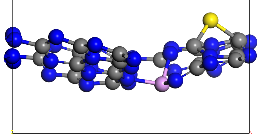 |
| 03 | 1P@C1 & S@Nbridge | 1.37 | 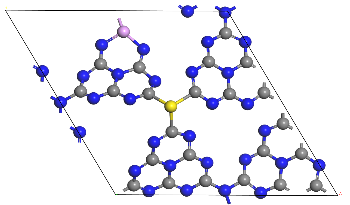 | 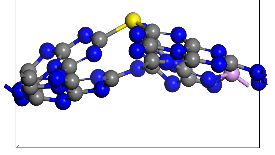 |
| 04 | 1P@C4 & S@N2 | -0.11 | 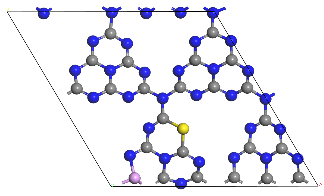 | 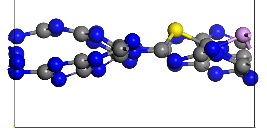 |
| 05 | 1P@C4 & S@N5 | -0.29 | 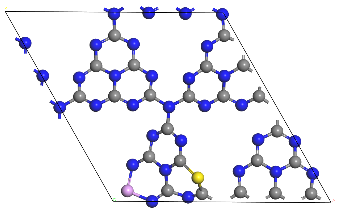 | 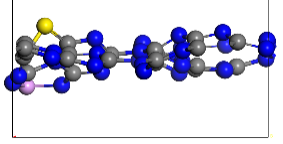 |
| 06 | 1P@C6 & S@N2 | -0.21 | 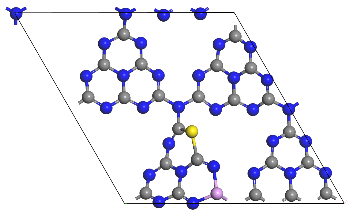 | 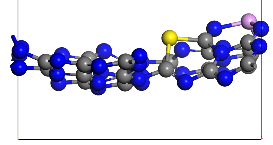 |

Table S2. Two P and one S co-doping configurations and formation energies of P-S:gCN in a monolayer

| **Sr.** | **Co-dopants sites** | **E_f_ (eV)** | **Structural Model** | |
| --- | --- | --- | --- | --- |
| **Two P and one S substitution sites** | | | **Top View** | **Side View** |
| 01 | 2P@C1 & S@N1 | 0.10 | 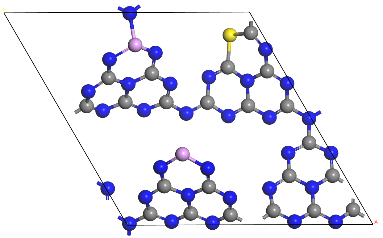 | 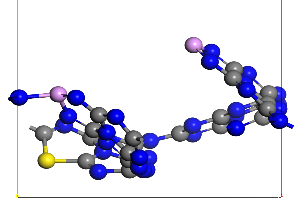 |
| 02 | 2P@C1 & S@N2 | -0.32 | 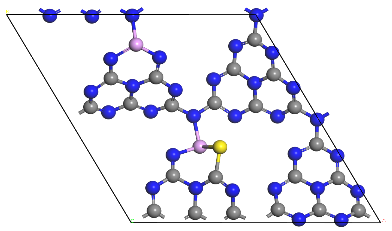 | 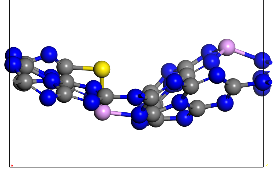 |
| 03 | 2P@C1 & S@N3 | -0.28 | 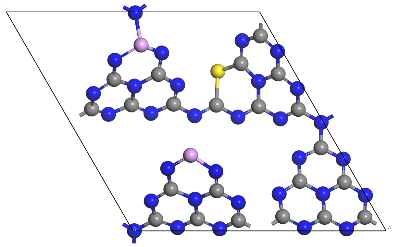 | 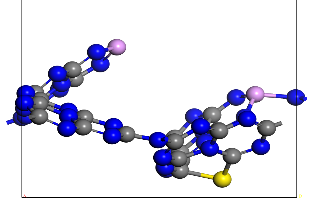 |
| 04 | 2P@C1 & S@N5 | 0.85 | 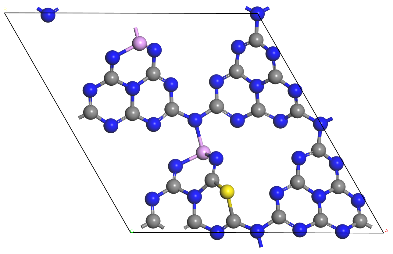 | 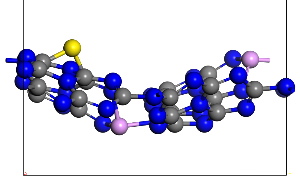 |
| 05 | 2P@C1 & S@N7 | 1.55 | 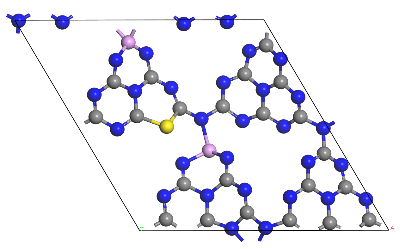 | 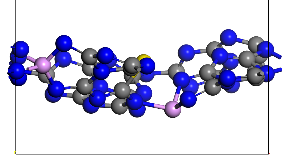 |
| 06 | 2P@C1,C4 & S@Nbridge | 2.18 | 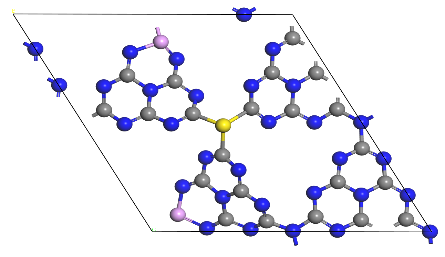 | 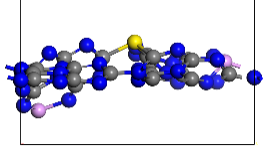 |

Table S3. One P and one S co-doping configurations and formation energies of P-S:gCN in bulk DFT models

| **Sr.** | **Co-dopants sites** | **E_f_ (eV)** | **Structural Model**  **Top View Side View** | |
| --- | --- | --- | --- | --- |
| 01 | 1P@C1 & S@N2 | -1.04 | 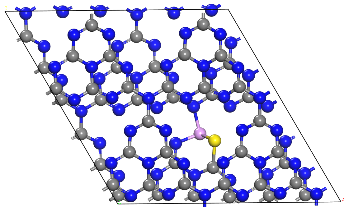 | 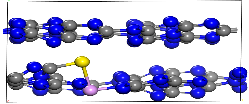 |
| 02 | 1P@C1 & S@N5 | -0.09 | 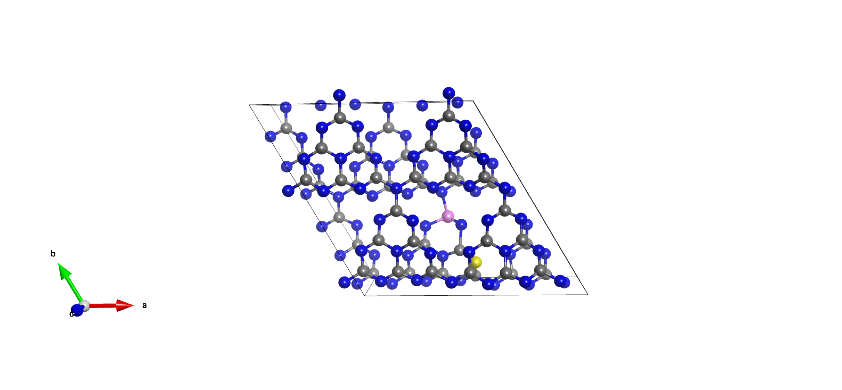 | **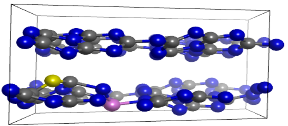** |
| 03 | 1P@C1 & S@Nbridge | 0.94 | **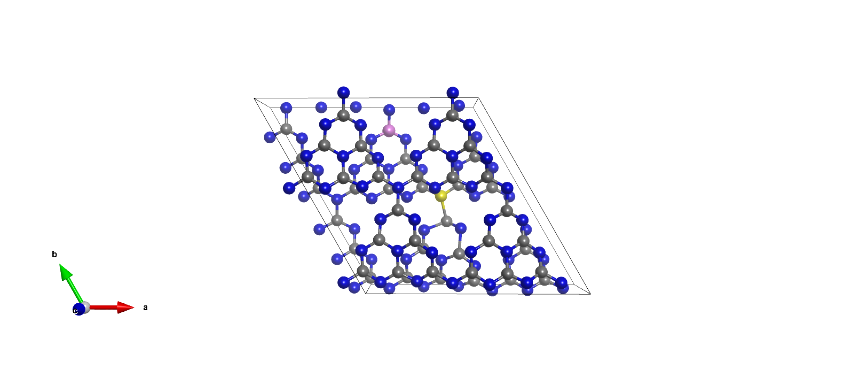** | 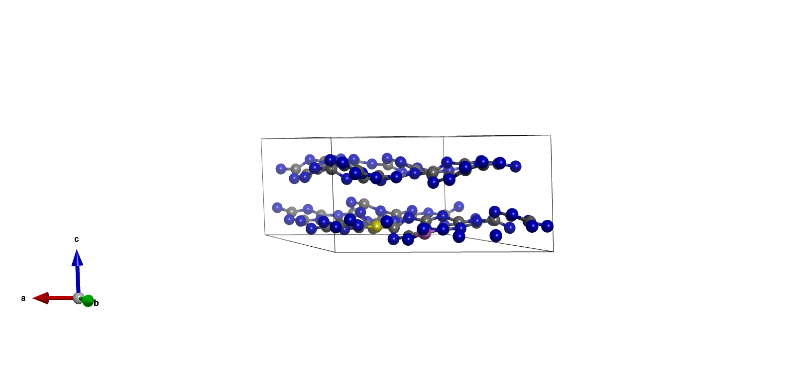 |
| 04 | 1P@C4 & S@N2 | -0.15 | 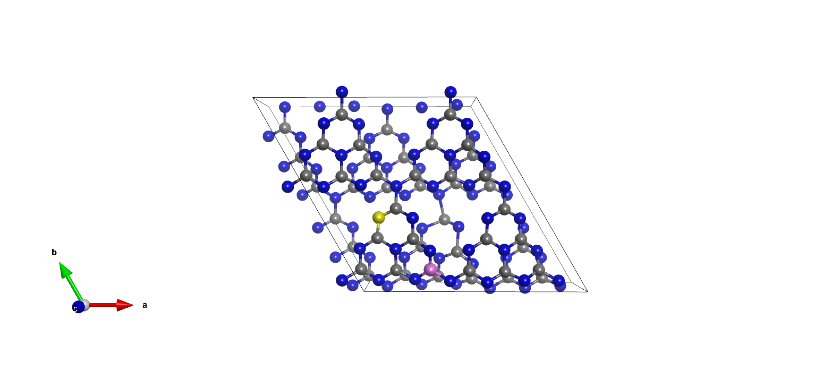 | 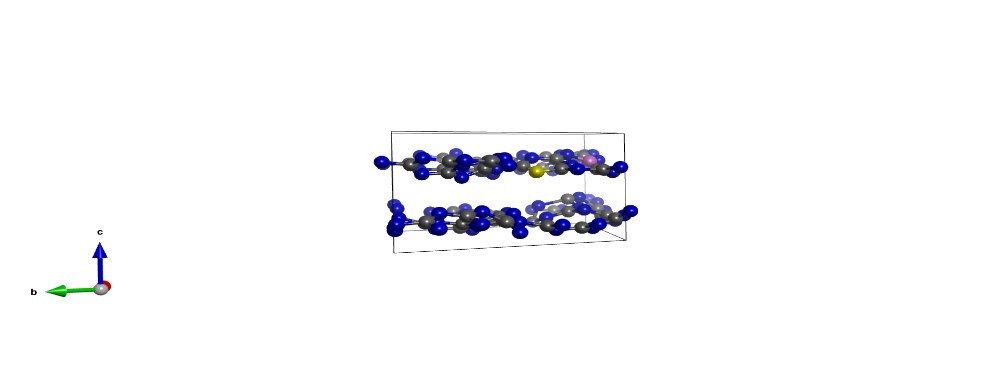 |
| 05 | 1P@C4 & S@N5 | -0.39 | 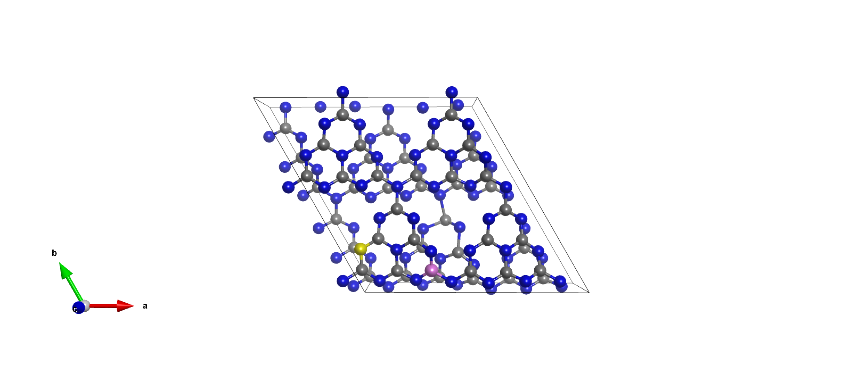 | 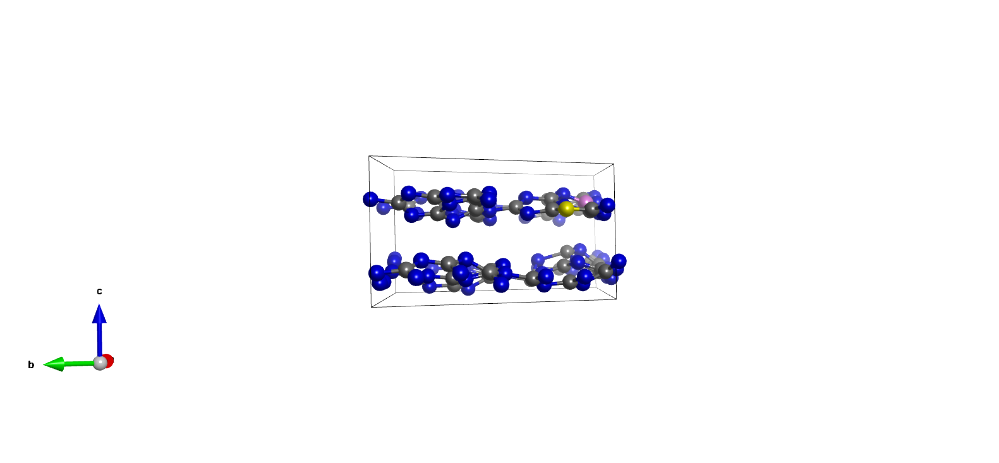 |


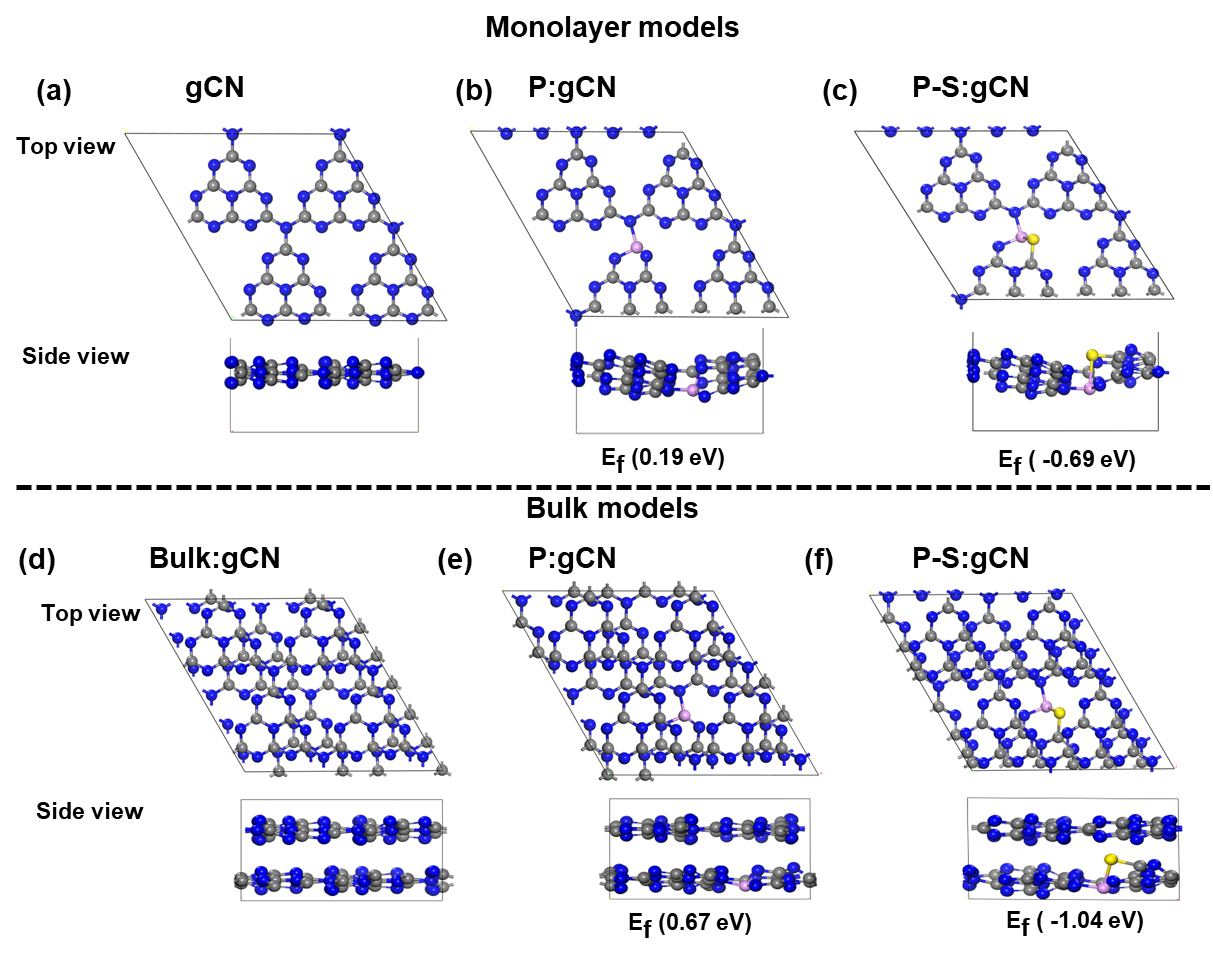


Figure S3. Optimized structural models of monolayer a-c) gCN, P:gCN, and P-S:gCN. Optimized structural models of d-f) Bulk:gCN, P:gCN, and P-S:gCN.

Table S4. Bader charge analysis for P:gCN and P-S:gCN

| Sample | Doped Atom | Bader charge transfer, ∆q (\|e\|) |
| --- | --- | --- |
| P:gCN | P@C1 site | +1.73 |
| P-S:gCN | P@C1 site  S@N2 site | +1.09  +0.19 |

**Interlayer spacing (d_002_) Calculation:**

**Bragg′s Equation** nλ = 2d.sinθ

For Bulk:gCN d_002_ = nλ/2sinθ

d_002_ = 0.15406/2sin(13.8)

**d_002_ = 0.323 nm**

For P-S:gCN d_002_ = nλ/2sinθ

d_002_ = 0.15406/2sin(13.6)

**d_002_ = 0.328 nm**


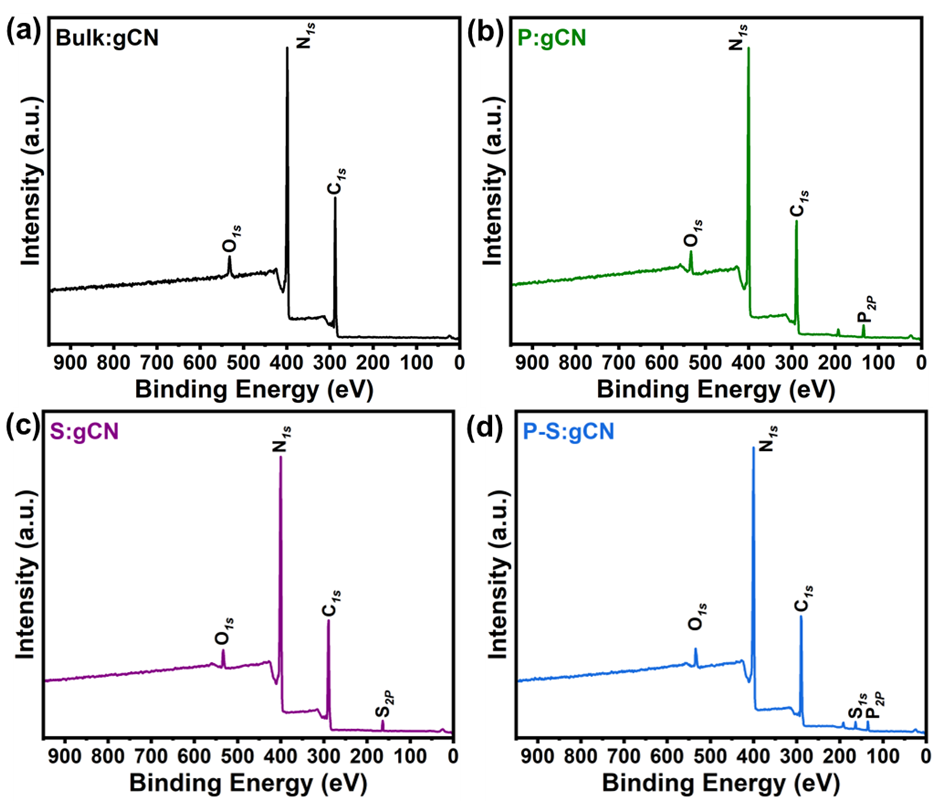


Figure S4. Survey XPS spectra of a) Bulk:gCN, b) P:gCN, c) S:gCN, d) P-S:gCN, respectively.


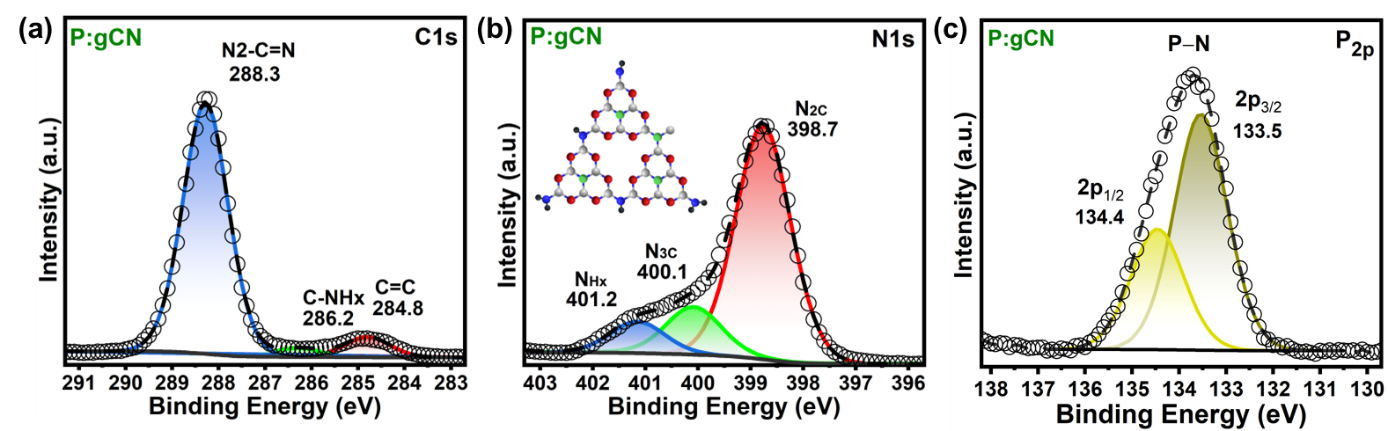


Figure S5. High-resolution C 1s, N1s, and P2p XPS spectra of P:gCN, respectively.

Table S5. Summary of the peak deconvolution from the high-resolution C 1s XPS spectra of the prepared samples

| **Sample** | **Peak area (%)** | | **Peak ratio of N-C=N and C-C** |
| --- | --- | --- | --- |
|  | **C-C** | **N-C=N** |  |
| **Bulk:gCN** | 8.66 | 91.34 | 10.54 |
| **P-S:gCN** | 10.01 | 89.98 | 8.98 |

Table S6. Element contents of Pristine gCN and co-doped gCN from the XPS analysis

|  | **Atomic Content (%)** | | | | | **Mass Content (%)** | |  |
| --- | --- | --- | --- | --- | --- | --- | --- | --- |
|  | **C** | **N** | **O** | **P** | **S** | **P** | **S** | |
| Bulk:gCN | 41.28 | 56.31 | 2.41 | - | - | - | - | |
| P:gCN | 38.23 | 56.36 | 2.37 | 3.04 | - | 6.82 |  | |
| S:gCN | 41.35 | 53.33 | 2.37 | - | 2.95 | - | 6.87 | |
| P-S:gCN | 39.74 | 54.77 | 2.42 | 1.56 | 1.52 | 3.50 | 3.53 | |

The mass content of P with respect to atomic content, specifically in samples P-S:gCN, is calculated as follows

**Mass content of P (%) =**

[(Atomic content of P (%) $\times$ atomic mass (g) of P) / {(Atomic content of C (%) $\times$ atomic mass (g) of C) + Atomic content of N (%) $\times$ atomic mass (g) of N) + (Atomic content of O (%) $\times$ atomic mass (g) of O) + (Atomic content of P (%) $\times$ atomic mass (g) of P) + (Atomic content of S (%) $\times$ atomic mass (g) of S)}]

**Mass content of P (%) = 3.50**

(1.56% $\times$ 30.97) / {(39.74% $\times$ 12.01) + (54.77% $\times$ 14.01) + (2.42% $\times$ 16.00) + (1.56% $\times$ 30.97) +(1.52% $\times$ 32.07)}

**Mass content of S (%) =**

[(Atomic content of S (%) $\times$ atomic mass (g) of S) / {(Atomic content of C (%) $\times$ atomic mass (g) of C) + Atomic content of N (%) $\times$ atomic mass (g) of N) + (Atomic content of O (%) $\times$ atomic mass (g) of O) + (Atomic content of P (%) $\times$ atomic mass (g) of P) + (Atomic content of S (%) $\times$ atomic mass (g) of S)}]

**Mass content of S (%) = 3.53**

(1.52% $\times$ 32.07) / {(39.74% $\times$ 12.01) + (54.77% $\times$ 14.01) + (2.42% $\times$ 16.00) + (1.56% $\times$ 30.97) +(1.52% $\times$ 32.07)}


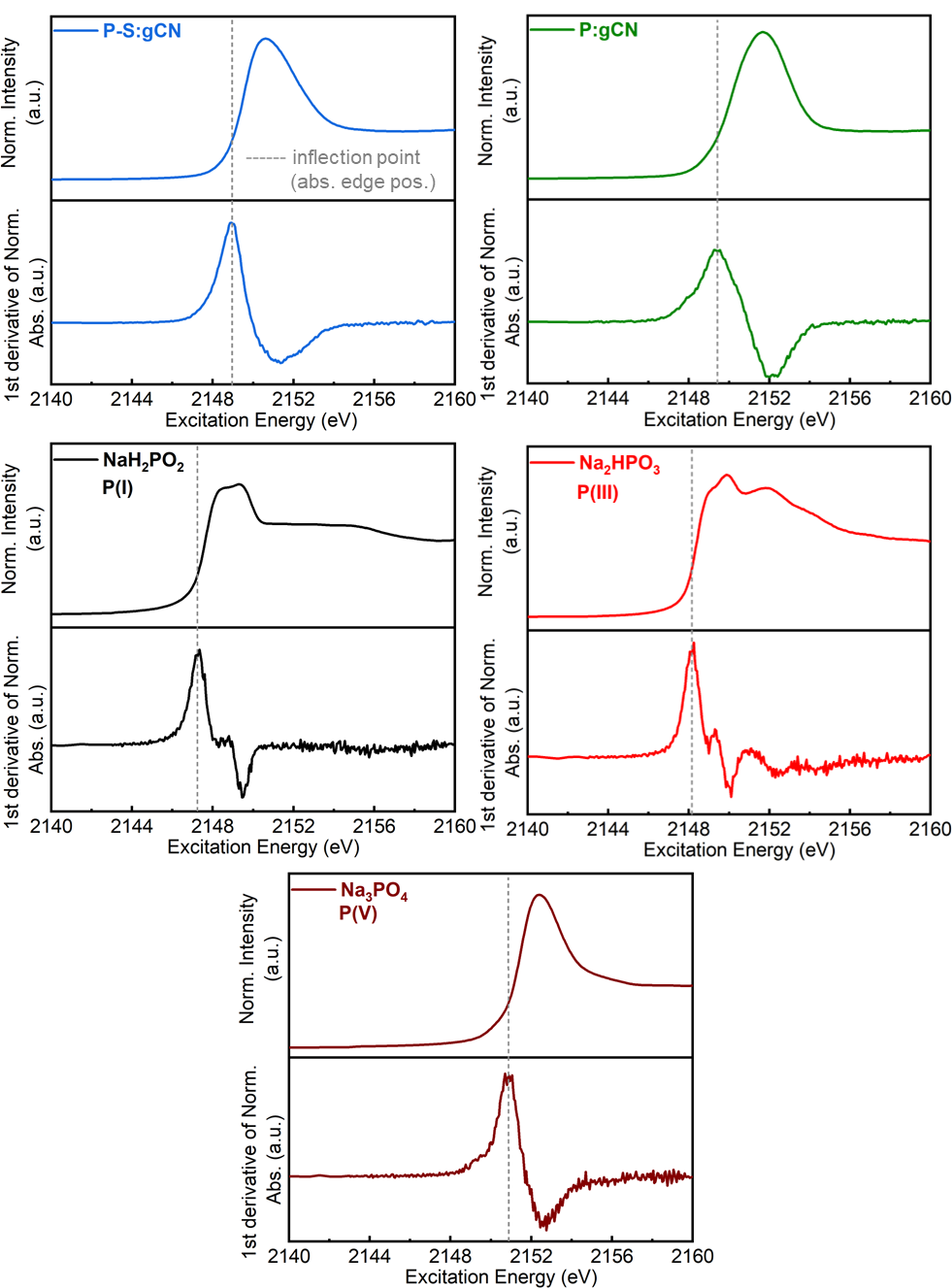


Figure S6. P K-edge XANES of phosphorus-containing compounds (top panels) and the 1st derivative of the compounds’ absorption spectra (bottom panels). Dashed lines represent the derived inflection points (considered to represent the absorption-edge).


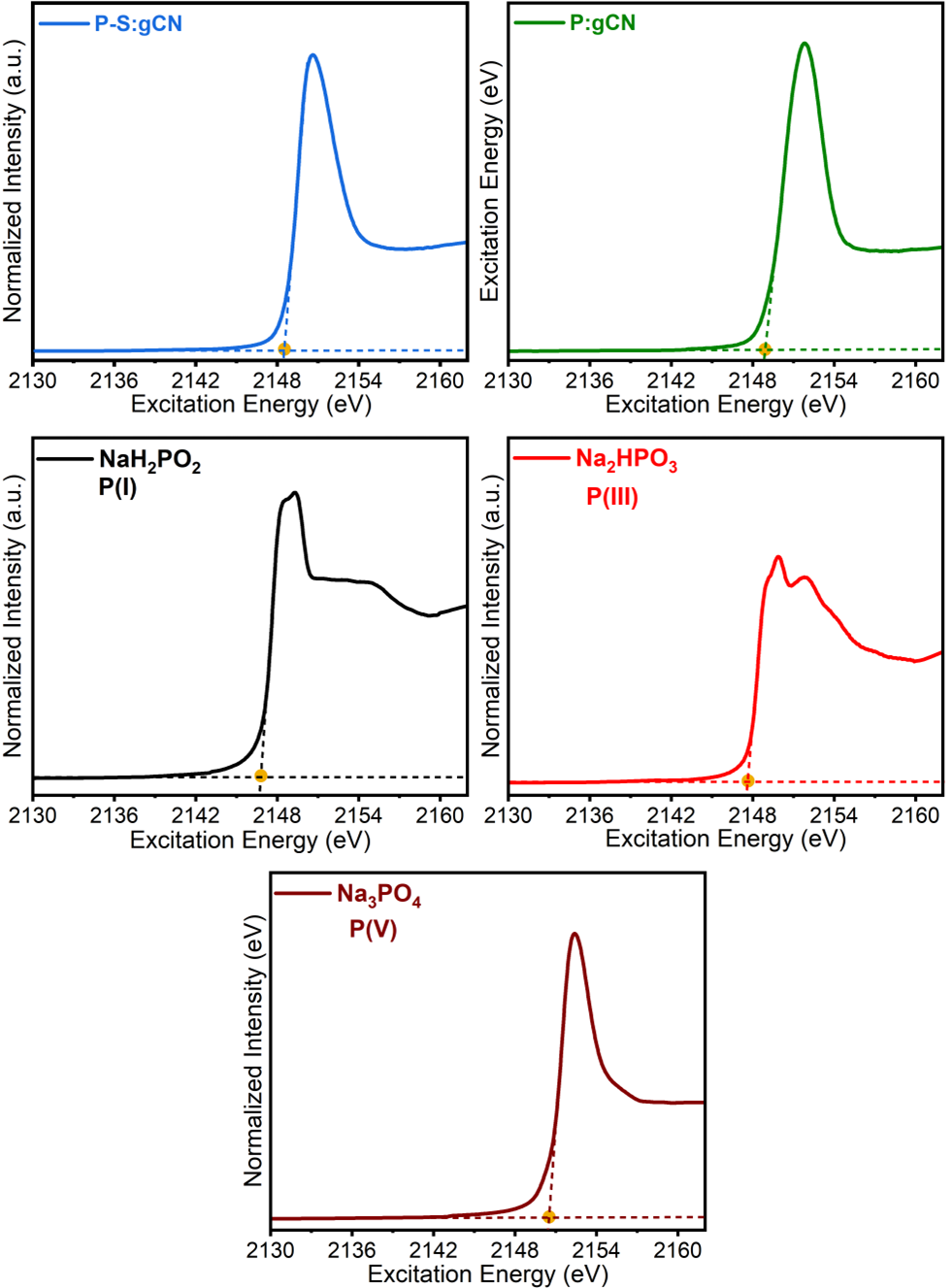


Figure S7. P K-edge XANES of P-containing compounds and the respective absorption-edge position through the intersection of the XANES background with the linear extrapolation of the rising edge of the 1st absorption maxima.

Table S7. P K-edge XANES absorption-edge positions and the respective uncertainties of the samples, as derived from P K-edge XANES data (Figure S6 and S7)

| **P compounds** | **P K-edge absorption-edge position (eV) determined by** | | **Uncertainty of the P K-edge absorption-edge position**  **(eV)** |
| --- | --- | --- | --- |
|  | **Inflection point** | **Linear extrapolation** |  |
| NaH_2_PO_2_ | 2147.23 | 2146.82 | 0.41 |
| Na_2_HPO_3_ | 2148.16 | 2147.63 | 0.53 |
| Na_3_PO_4_ | 2150.89 | 2150.46 | 0.43 |
| P:gCN | 2149.41 | 2148.9 | 0.51 |
| P-S:gCN | 2148.95 | 2148.5 | 0.45 |

Table S8. DFT Proposed structural model validation through PDF analysis

| P-S:gCN model of atomically correlated P-S pair  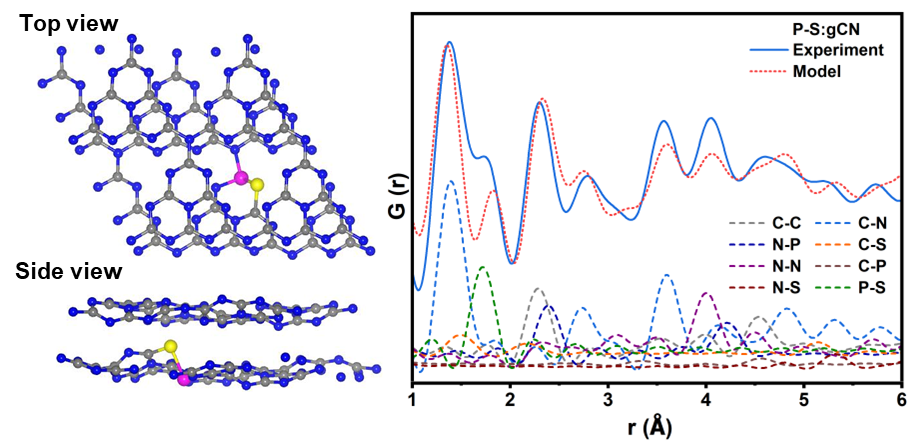 |
| --- |
| P-S:gCN model with 2P and 1S substitution  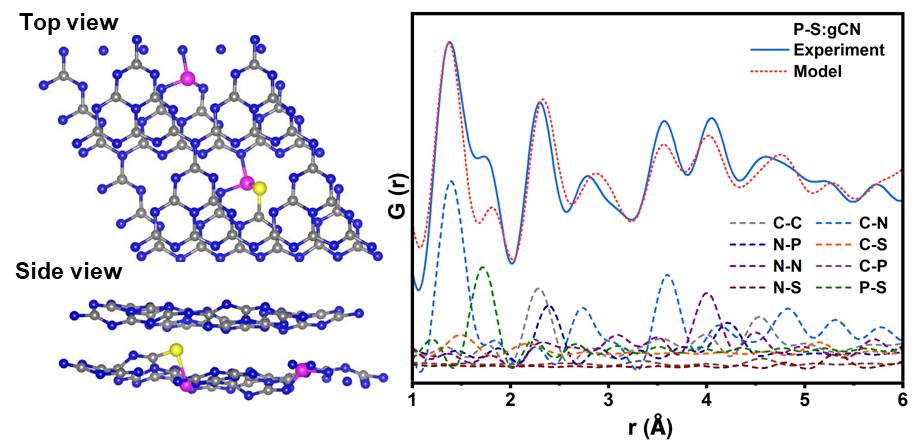 |
| P-S:gCN model with two pairs of atomically correlated P-S sites (Close to matched structure)  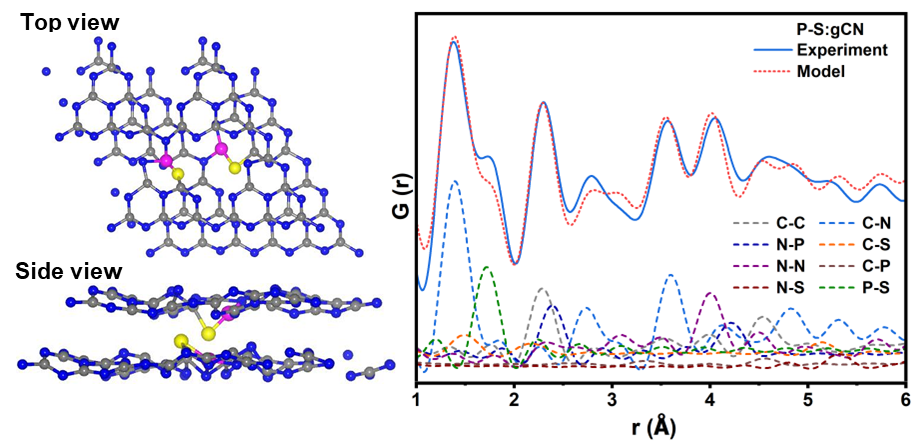 |

Grey : C, Blue : N, Yellow : S, Purple : P

| P-S:gCN model with 3P and 2S substitution  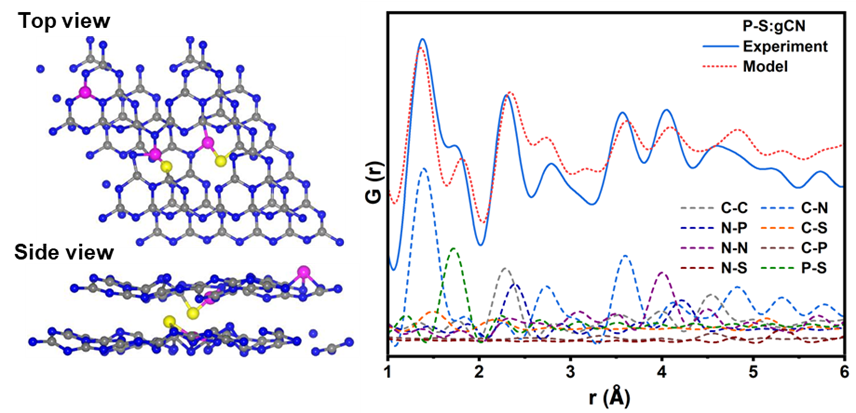 |
| --- |
| P-S:gCN model with three pairs of atomically correlated P-S sites  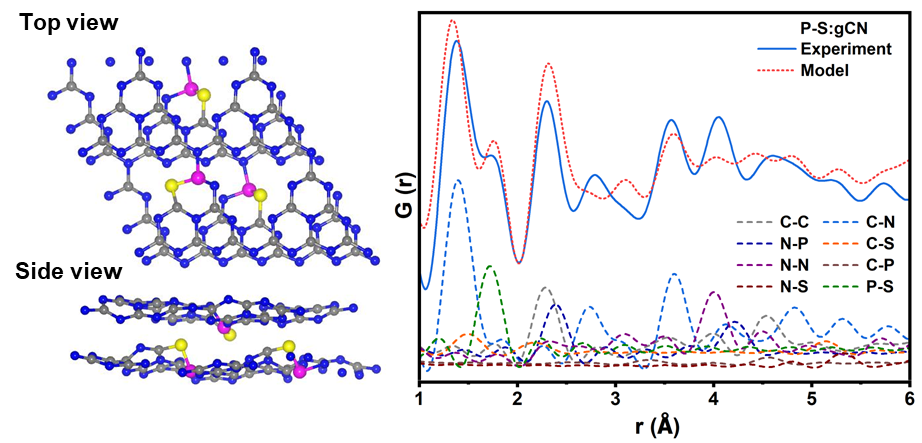 |
| P-S:gCN model with four pairs of atomically correlated P-S sites  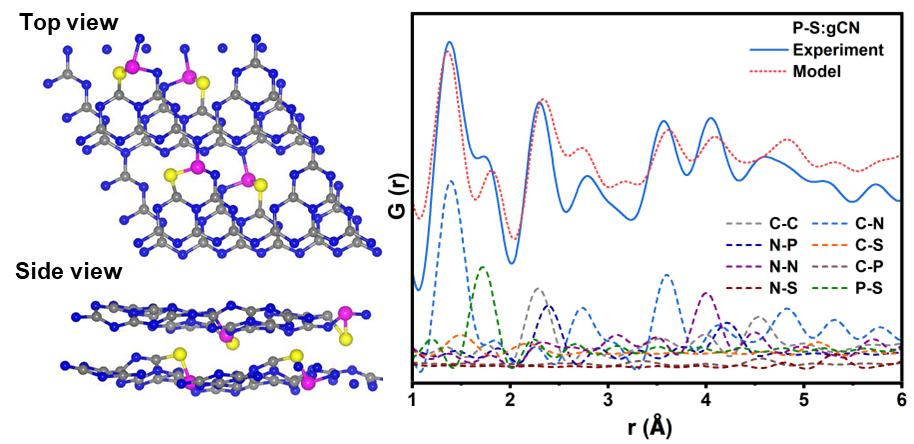 |

Table S9. Best matched DFT structural model with experiment, along with atomic coordinates.

| P-S:gCN model with two pairs of atomically correlated P-S sites (Best matched)  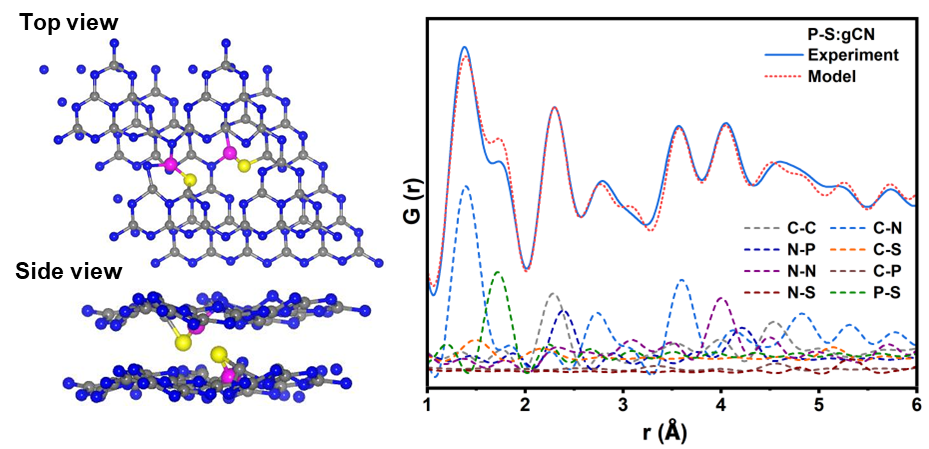 | | |
| --- | --- | --- |
| Atomic pair/ coordinates | **Distance r (Å)** | |
|  | **Bulk:gCN** | **P-S:gCN** |
| 1^st^ coordination (direct bonding) | | |
| C-N | 1.35 | 1.39 |
| P-N | - | 1.68 |
| C-S | - | 1.49 |
| P-S | - | 1.74 |
| 2^nd^ or higher coordination | | |
| C-C | 2.25 | 2.29 |
| C-N | 2.72 | 2.72 |
| N-N | 2.65 | 3.08 |
| C-P | - | 3.35 |
| C-S | - | 2.17 |
| N-P | - | 2.38 |
| N-S | - | 4.79 |
| P-S | - | 2.67 |


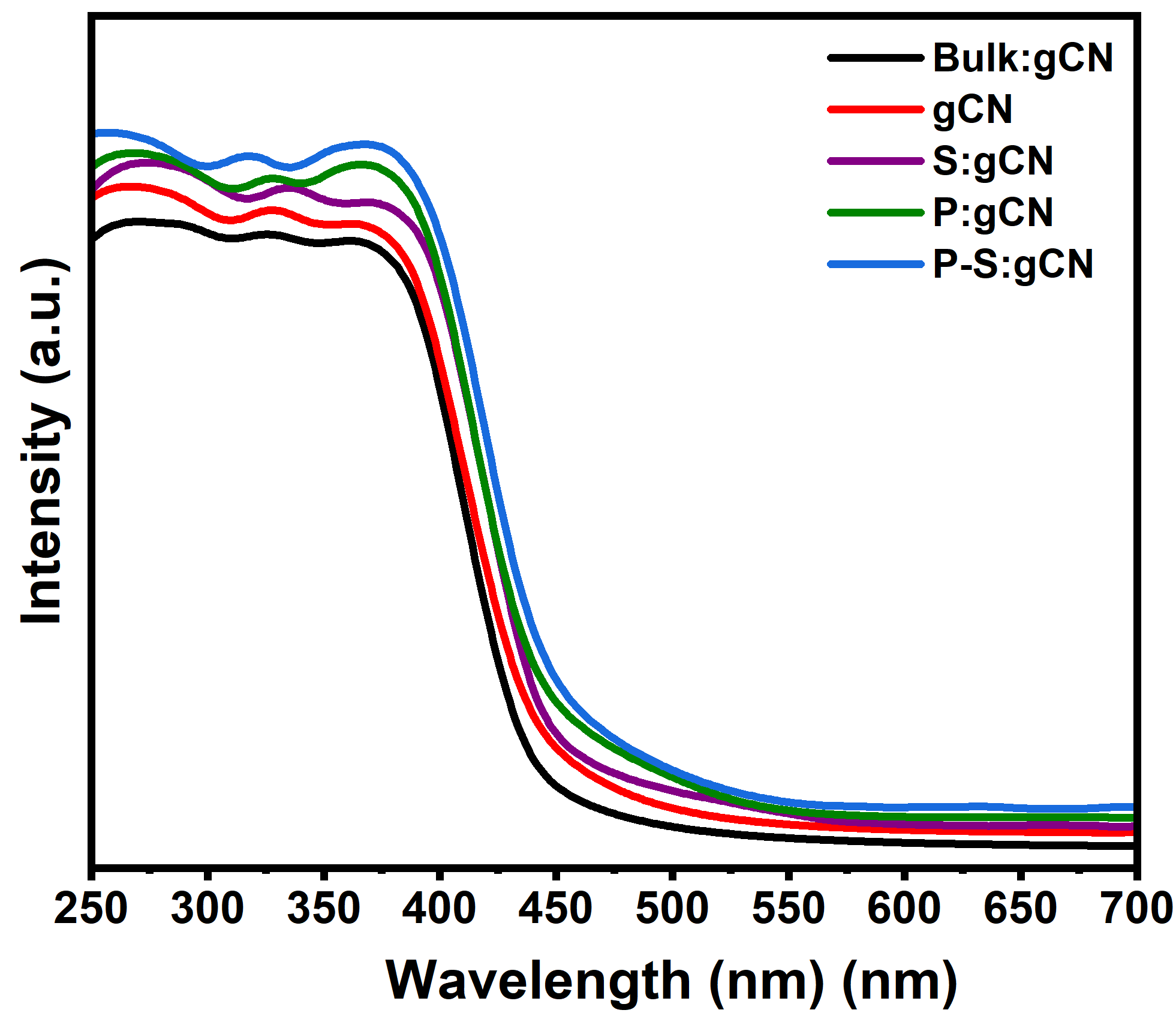


Figure S8. UV-Vis DRS of Bulk:gCN, gCN, S:gCN, P,gCN, and P-S:gCN.

Table S10. Average lifetimes of the as-prepared sample through TRPL.

| **Samples** | **τ_1_/ns (A_1_)** | **τ_2_/ns (A_2_)** | **τ_3_/ns (A_3_)** | **τ_A_/ns^a^** |
| --- | --- | --- | --- | --- |
| Bulk:gCN | 1.24 (18.09%) | 3.946 (48.87%) | 19.09 (33.04%) | 8.46 |
| gCN | 0.878 (30.25%) | 3.918 (40.19%) | 18.99 (29.56%) | 7.45 |
| S:gCN | 0.994 (40.57%) | 3.752 (36.25%) | 15.46 (23.18%) | 5.35 |
| P;gCN | 0.654 (39.86%) | 3.523 (38.75%) | 15.21 (21.39%) | 4.88 |
| P-S:gCN | 0.728 (52.04%) | 3.367 (34.46%) | 13.31 (13.5%) | 3.34 |

The average TRPL lifetime is calculated by using the equation. $\tau_{A}=\frac{\sum A_{i}\tau_{i}}{\sum A_{i}}$ , where $\tau_{i}$ is the time coefficient and $A_{i}$ is the corresponding amplitude of each component.


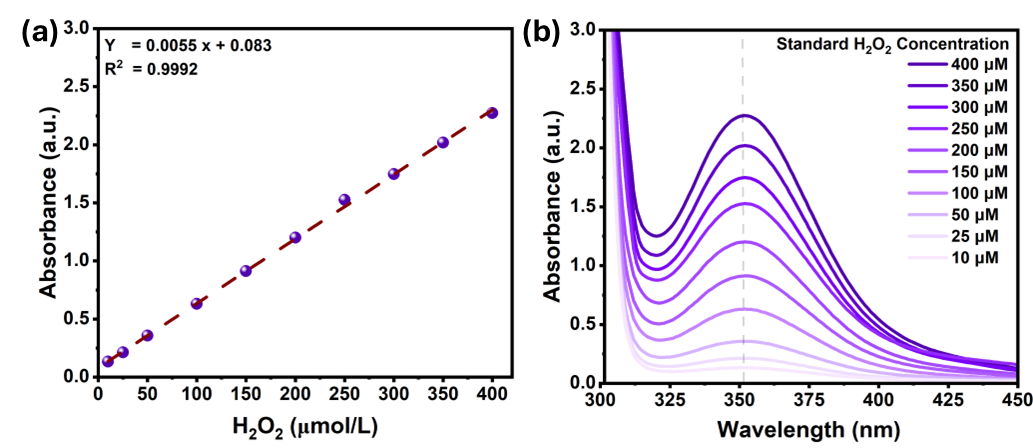


Figure S9. a) Linear relationship between absorbance and H₂O₂ concentration. b) Corresponding UV–Vis absorption spectra of standard H₂O₂ solutions at increasing concentrations, showing a characteristic peak at ~350 nm used for quantification.


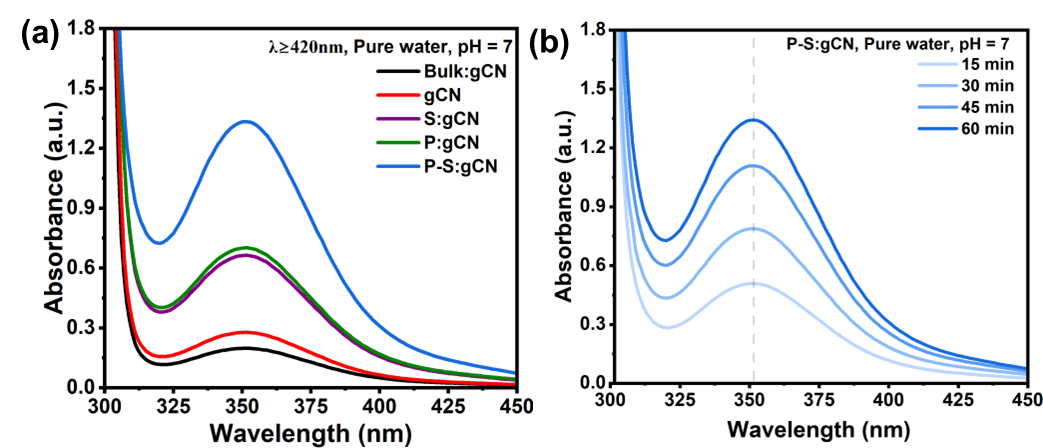


Figure S10. Photocatalytic production of H₂O₂ assessed using UV–Vis spectroscopy. a) Comparison of UV-Vis absorbance among the prepared gCN photocatalysts for H₂O₂ detection at 350 nm. b) Time-dependent H₂O₂ generation of P–S:gCN under visible-light irradiation in pure water at pH 7


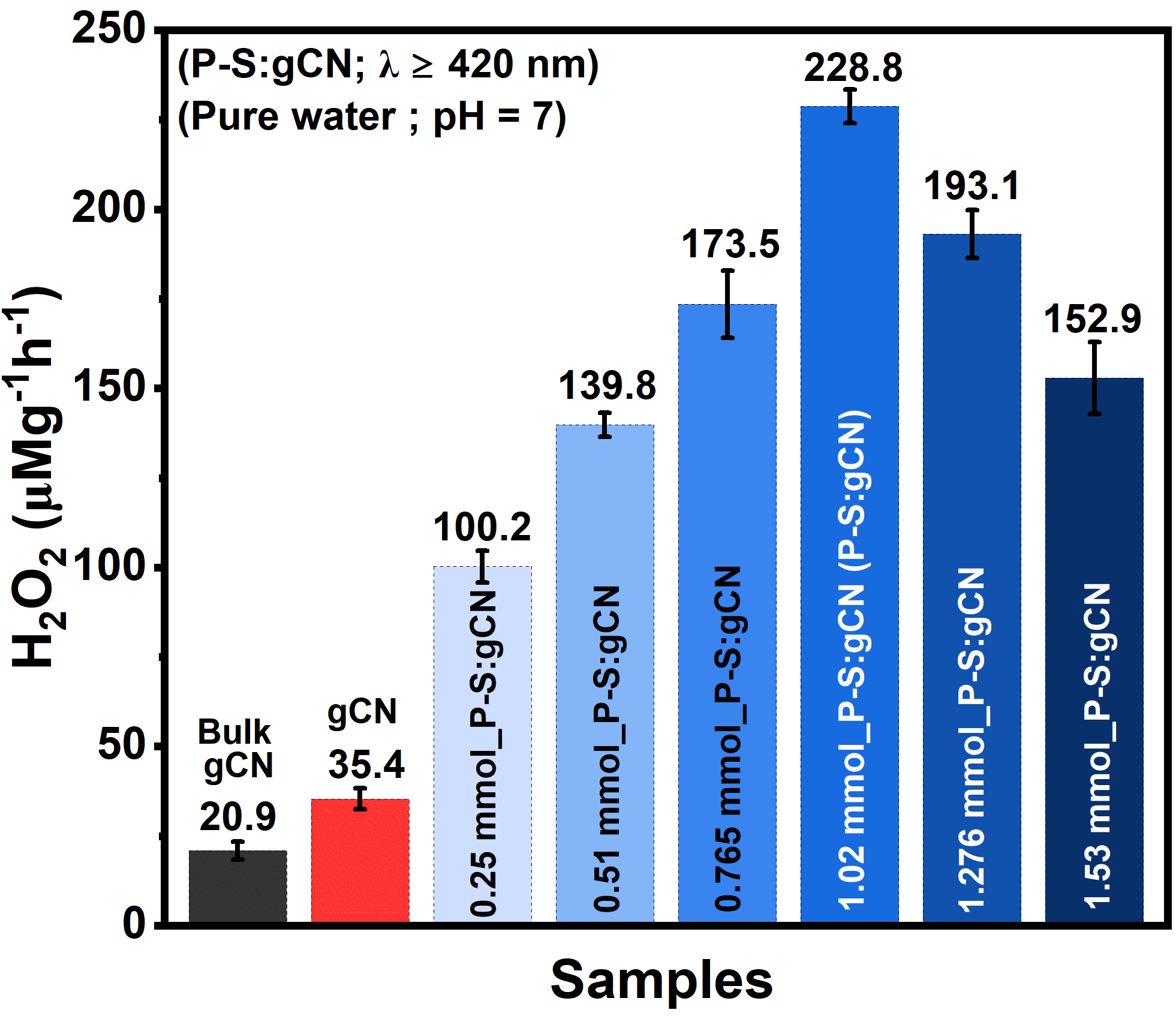


Figure S11. H₂O₂ production rates of P–S co-doped g-C₃N₄ samples with different concentrations of dopants.

## Dopant loading for comparative samples

For all comparative studies among S:gCN, P:gCN, and P-S:gCN, the total dopant concentration was carefully controlled to ensure meaningful comparisons. In the singly doped samples (P:gCN or S:gCN), the molar amount of the respective dopant precursor was set equal to the total dopant content in P-S:gCN.

Accordingly, comparisons between singly doped and co-doped samples were made at comparable overall dopant levels (approximately 7 wt%) rather than identical precursor masses. This approach ensures that observed differences in electronic structure, charge-carrier dynamics, and photocatalytic performance primarily arise from the dopant-correlated environment (isolated vs P-S paired sites) rather than from variations in dopant concentration.

In this context, P-S:gCN contains a similar total number of dopant sites as P:gCN or S:gCN, but these sites are predominantly paired as atomically correlated P-S motifs, whereas P:gCN and S:gCN contain isolated P or S-centred sites, respectively.


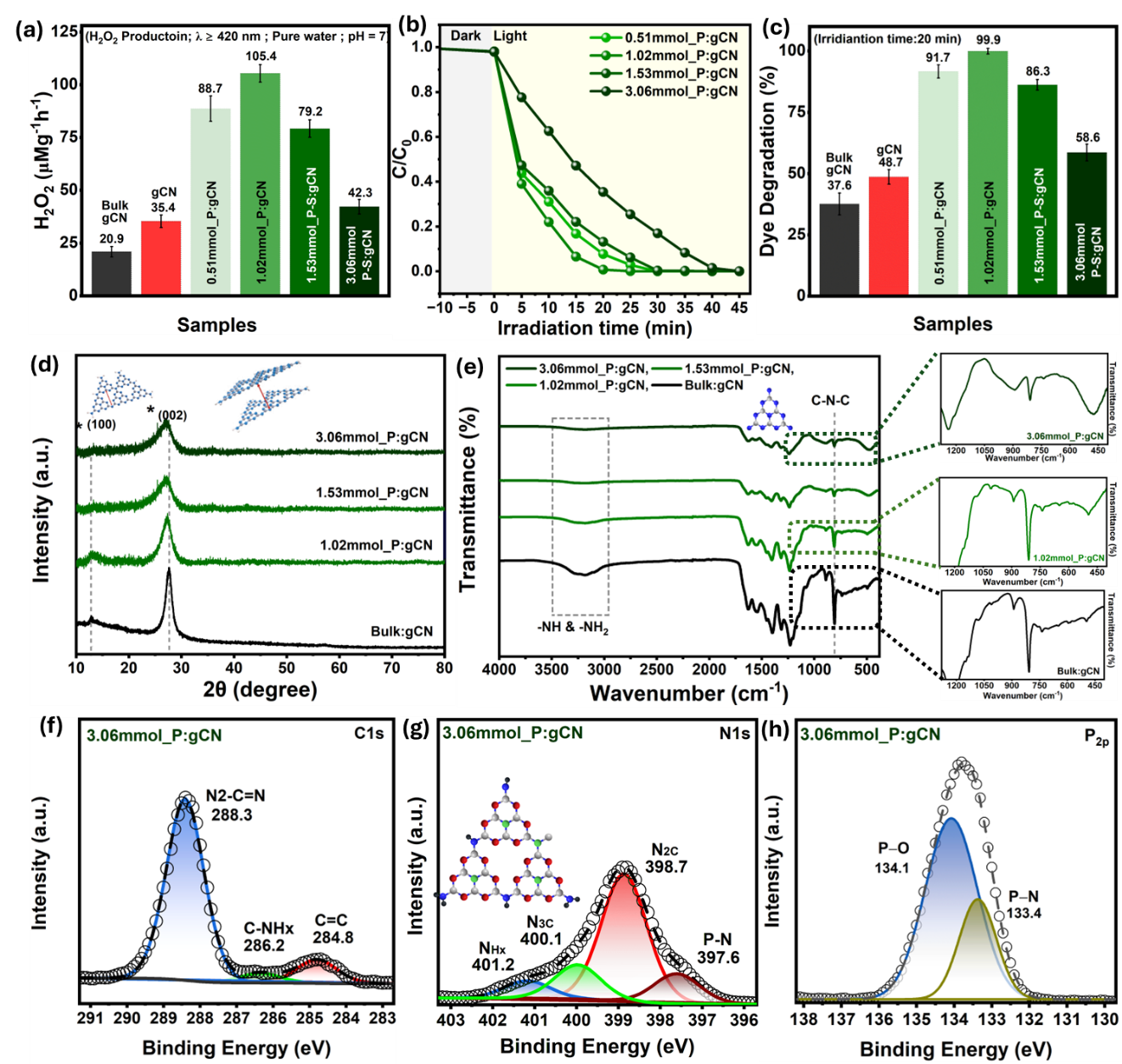


Figure S12. a) H_2_O_2_ production rates of bulk:gCN and P:gCN with varying P loading under λ ≥ 420 nm irradiation in pure water (pH 7). b) Photocatalytic dye degradation profiles under visible light. c) Corresponding dye-degradation efficiencies after 20 min irradiation. d-e) XRD patterns and FTIR with different P content in g-C_3_N_4_. e) (f–h) High-resolution XPS spectra of C 1s, N 1s, and P 2p regions for the 3.06 mmol P-gCN sample, confirming the preservation of the C-N heterocycles, the emergence of P-N coordination, and the presence of P-O species originating from partially oxidized P sites.

Table S11. AQY of H₂O₂ generation over P-S:gCN at different monochromatic wavelengths.

| Wavelength (nm) | 380 | 400 | 420 | 450 | 500 | 550 |
| --- | --- | --- | --- | --- | --- | --- |
| H_2_O_2_ formed (µmol) | 19.77 | 11.95 | 9.89 | 3.04 | 1.18 | 0.40 |
| Light intensity (mW) | 26.58 | 22.10 | 23.00 | 20.15 | 21.27 | 19.68 |
| Irradiation area (cm^2^) | 1.70 | 1.70 | 1.70 | 1.70 | 1.70 | 1.70 |
| Irradiation time (h) | 1 | 1 | 1 | 1 | 1 | 1 |
| AQY (%) | 13.01 | 8.99 | 6.80 | 2.23 | 0.74 | 0.25 |

**
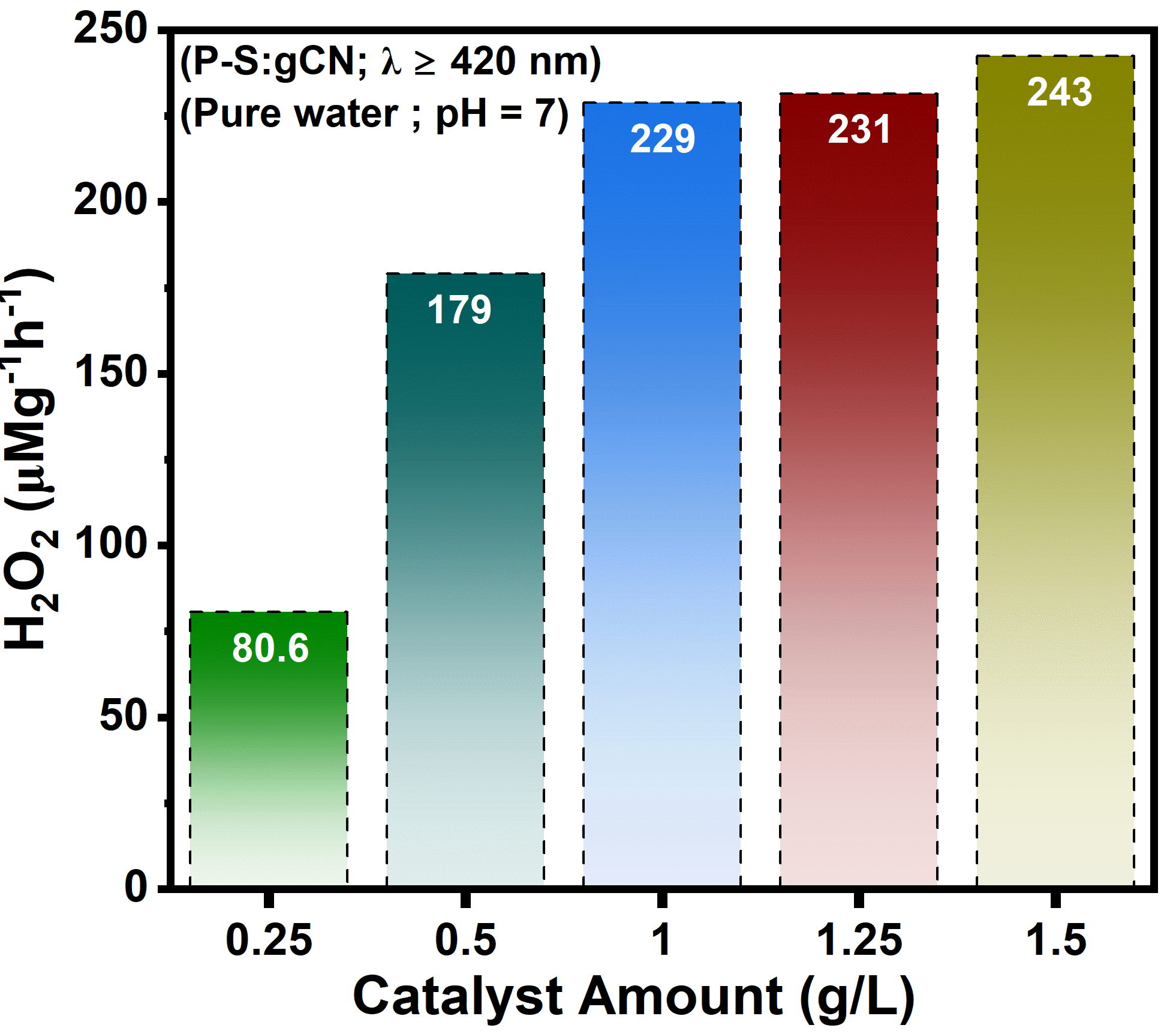
**

Figure S13. Effect of catalyst loading on photocatalytic H₂O₂ production over P–S:gCN under visible-light irradiation (λ ≥ 420 nm) in pure water at pH 7.

The H₂O₂ generation rate increases with catalyst concentration from 0.25 to 1.5 g·L⁻¹, reaching a maximum of 243 μmol g⁻¹ h⁻¹ at 1.5 g·L⁻¹. The enhancement is attributed to the increased number of active sites available for the two-electron oxygen reduction reaction (2e⁻ ORR). However, the rate of increase diminishes at higher loadings, possibly due to light scattering or limited photon penetration.


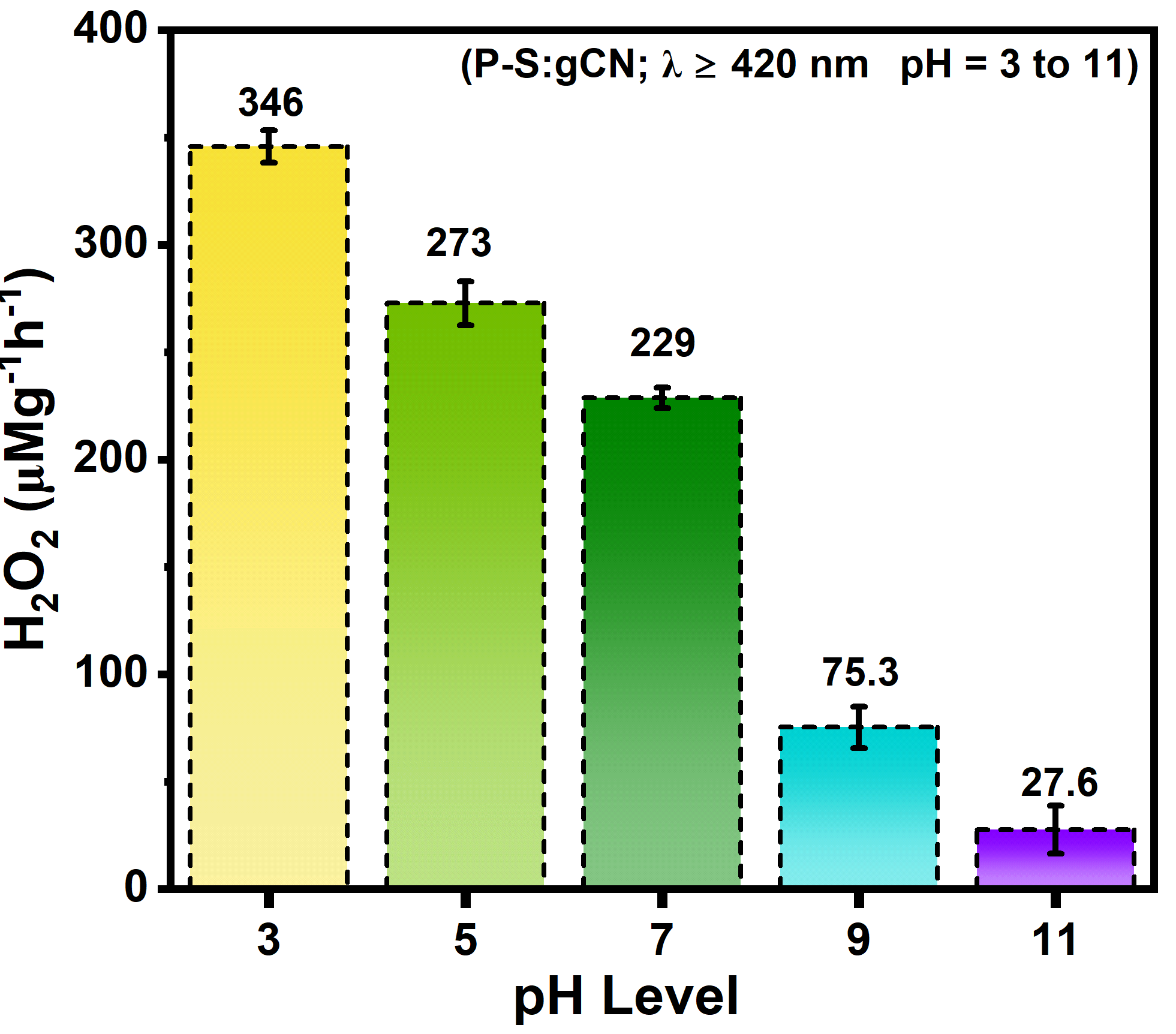


Figure S14. Effect of pH on photocatalytic H₂O₂ production using P–S:gCN under visible-light irradiation (λ ≥ 420 nm).

The H₂O₂ production efficiency of P-S:gCN strongly depends on pH, with the highest yield at acidic pH 3 (346 μmol g⁻¹ h⁻¹). This enhanced performance in acidic conditions is attributed to the increased proton availability, which supports the 2e⁻ ORR and promotes the selective formation of H₂O₂. As the pH rises, the proton concentration decreases, weakening the thermodynamic drive for the ORR and increasing the likelihood of side reactions or H₂O₂ decomposition, especially at alkaline pH levels (75.3 μmol g⁻¹ h⁻¹ at pH 9 and only 27.6 μmol g⁻¹ h⁻¹ at pH 11). Additionally, under basic conditions, H₂O₂ is less stable due to rapid base-catalyzed decomposition and possible radical-mediated breakdown, further reducing its accumulation. The optimal H₂O₂ generation near pH 3-5 aligns with favourable proton-coupled electron transfer kinetics and the suppression of over-oxidation pathways.


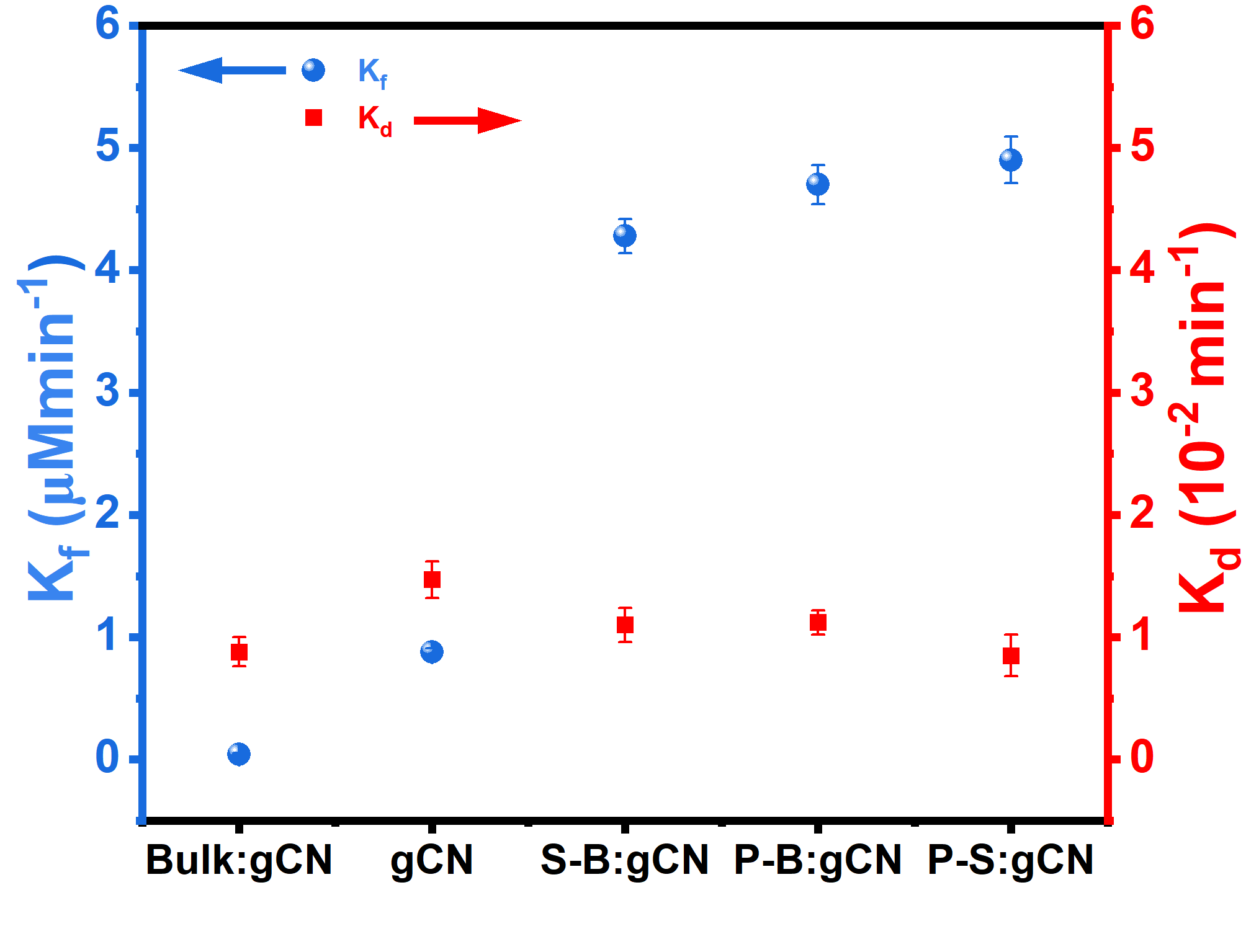


Figure S15. Kinetic analysis of H₂O₂ formation (K_f_) and decomposition (K_d_) over as-prepared g-C_3_N_4_ photocatalysts under visible light (λ ≥ 420 nm).

The kinetic parameters K_f_ and K_d_ were measured to evaluate the balance between H₂O₂ formation and decomposition. Bulk:gCN and gCN show the lowest H₂O₂ production rates and the highest decomposition rates, indicating poor catalytic selectivity and stability for H₂O₂ generation. With modifications, especially involving co-dopants, the formation rates increase significantly. Among all samples, P-S:gCN exhibits the highest H₂O₂ formation rate (K_f_ 4.9 μM·min⁻¹) and the lowest decomposition rate (K_d_ 0.85 × 10⁻² min⁻¹), demonstrating its superior catalytic activity and stability. This is due to the synergistic P-S correlation, which promotes selective 2e⁻ ORR while limiting over-oxidation or degradation pathways.


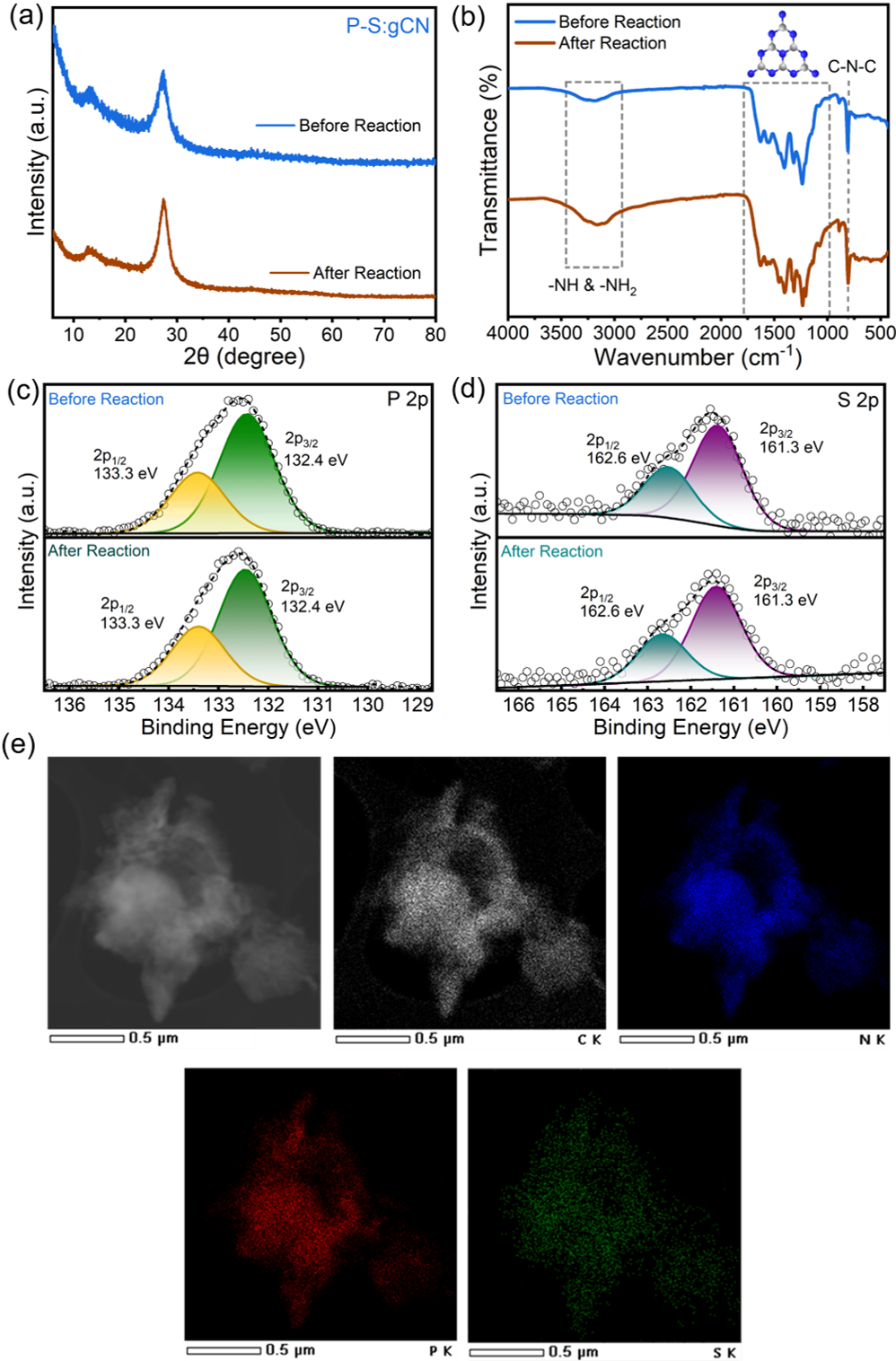


Figure S16. a) XRD patterns, b) FTIR spectra, and c, d) High resolution spectra of P 2p and S 2p, (e) elemental mapping images (C, N, P, S) of P-S:gCN before and after photocatalytic stability reaction for 24 h.

Table S12. Element contents of P-S:gCN before and after 24 h of photocatalytic reaction

|  | **Atomic Content (%)** | | | | | **Mass Content (%)** | | |  |
| --- | --- | --- | --- | --- | --- | --- | --- | --- | --- |
|  | **C** | **N** | **O** | **P** | **S** | | **P** | **S** | |
| Before reaction | 39.74 | 54.77 | 2.41 | 1.56 | 1.52 | | 3.50 | 3.53 | |
| After reaction | 39.78 | 54.82 | 2.37 | 1.55 | 1.50 | | 3.48 | 3.49 | |


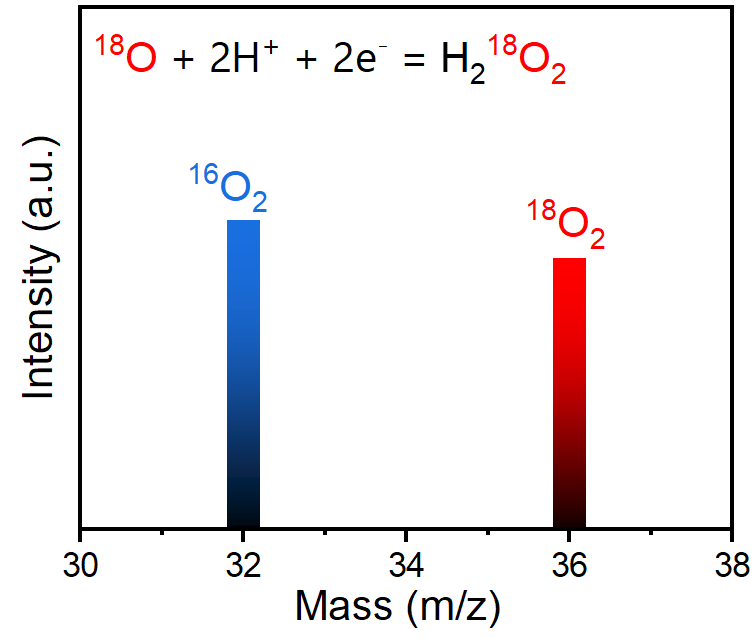


Figure S17. Isotope-labelling mass spectrometric analysis conducted in H₂¹⁶O under a ¹⁸O₂ atmosphere.


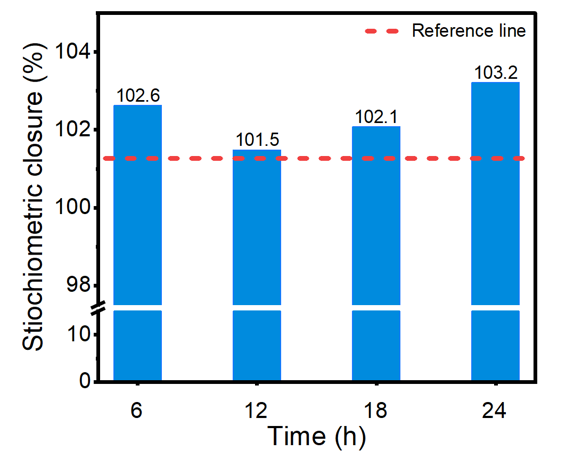


Figure S18. Stoichiometric closure, calculated as the ratio of the measured to the theoretical O₂ decrease, at different irradiation times.


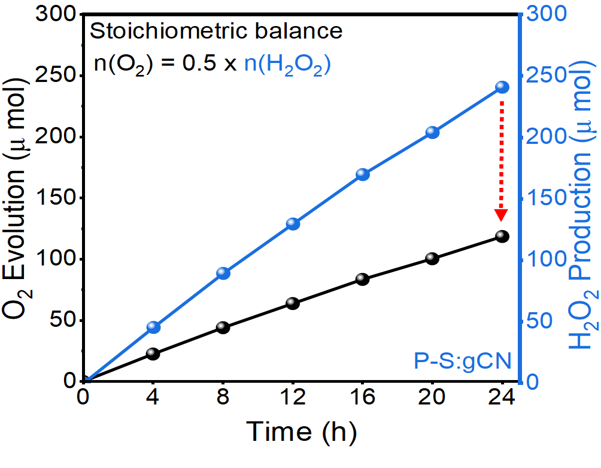


Figure S19. Time-dependent O₂ evolution and H₂O₂ production


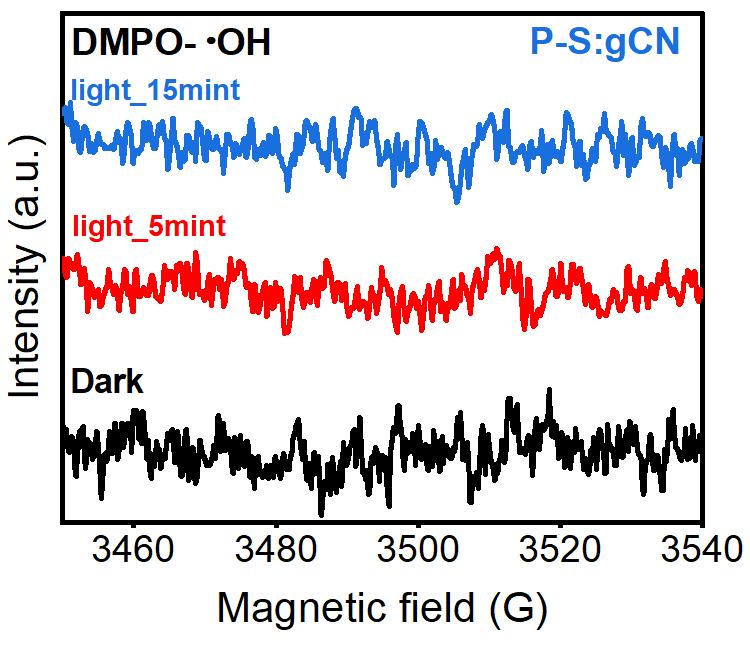


Figure S20. Time-dependent EPR spectra of the DMPO-·OH spin adduct over P-S:gCN under dark conditions and visible-light irradiation for 5 and 15 min.

Table S13. SCC of as-prepared photocatalysts

| **Sample** | **ΔGH_2_O_2_**  **(kJ mol^-1^)** | **light intensity (mW.cm^-2)^** | **Irradiation area (cm^2^)** | **Irradiation time (h)** | **H_2_O_2_ (μmol)** | **SCC**  **%** |
| --- | --- | --- | --- | --- | --- | --- |
| P-S:gCN | 117 | 100 | 1.7 | 3 | 76.94 | 0.49 |

$$\mathrm{SCC}=\frac{\left[ {\Delta G}_{H_{2}O_{2}} \right]\times\left[ n_{H_{2}O_{2}} \right]}{I\times S\times T}\times100\%= \frac{117 kJ{mol}^{-1}\times76.94 \mu mol}{100mW{cm}^{-2}\times1.7{cm}^{2}\times10800s}\times100\%=0.49\%$$

Table S14. Summary of g-C_3_N_4_-based materials for photocatalytic H_2_O_2_ generation activity.

| **Catalysts** | **Sacrificial Reagent** | **Light Source** | **H_2_O_2_ yield (μmolg^-1^h^-1^)** | **AQY%**  **at 420 nm** | **SCC**  **%** | **Ref** |
| --- | --- | --- | --- | --- | --- | --- |
| P-S:gCN | Pure water | λ≥420 nm | 229 | 6.8% | 0.5 (3h) | This work |
|  |  | AM 1.5G | 363 | - | - |  |
| Ph-MCN | Pure water | λ≥420 nm | 195 | - | - | [3] |
| Na-PHI | Pure water | λ ≥380 nm | 155 | 1.9% | - | [4] |
| K5-CN | Pure water | λ≥420 nm | 115 | - | - | [5] |
| M-CNT | Pure water | λ≥420 nm | 44 | 2.3% | - | [6] |
| CNOP | IPA 10% | λ≥420 nm | 255 | - | - | [7] |
| TA-CN-5 | Pure water | λ≥420 nm | 142 | 3.2% | - | [8] |
| s-B0.5CN | Pure water | λ≥420 nm | 67 | - | - | [9] |
| C_3_N_4_-nv | Pure water | λ≥420 nm | 98 | 1.9% | - | Lu, et al. ^[10]^ |
| gC_3_N_4_-TP | IPA 10% | λ≥420 nm | 552 | 3.2% | - | [11] |
| PCN-D-1 | Pure water | λ≥420 nm | 52 | - | - | [12] |
| [CDs@CTFs](mailto:CDs@CTFs) | Pure water | λ≥420 nm | 78 | 0.9% | 0.2 (3h) | [13] |
| Nv-C≡N-CN | Pure water | λ≥420 nm | 137 | 1.8% | 0.2 (3h) | [14] |
| RP/GCN | Pure water | λ≥420 nm | 125 | - | - | [15] |
| [5Cv@g-C_3_N_4_](mailto:5Cv@g-C3N4) | Pure water | λ≥420 nm | 125 | - | - | [16] |
| CNK0.2 | 5% methanol | λ≥420 nm | 101 | - | - | [17] |
| g-CN-MI-40 | Pure water | λ≥420 nm | 120 | - |  | [18] |
| C-N-g-C_3_N_4_ | Pure water | 420nm  ≤λ≥ 700nm | 12 | - | - | [19] |
| OCN-500 | Pure water | λ≥420 nm | 5.3 | - | - | [20] |
| R370-CN | Pure water | λ≥420 nm | 170 | 4.3% | 0.3 (3h) | [21] |
| C_3_N_4_/PDI/rGO | Pure water | λ≥420 nm | 290 | - | 0.2 (3h) | [22] |
| PI0.5-NCN | Pure water | λ≥420 nm | 101 | 3.2% | - | [23] |
| g-C_3_N_4_/PDI | Pure water | λ≥420 nm | 50.1 | 2.7% | - | [24] |

Table S15. Time-dependent stoichiometric balance of O_2_ through WOR and ORR

| **Time**  **(h)** | **H₂O₂ produced (µmol)** | **Expected O₂ decrease**  **= 0.5×n(H_2_​O_2​_) (µmol)** | **Measured**  **O₂ drop (µmol)** | **Stoichiometric Closure (%)** |
| --- | --- | --- | --- | --- |
| 6 | 64.3 | 32.1 | 32.9 | 102.6 |
| 12 | 120.9 | 60.4 | 61.3 | 101.5 |
| 18 | 173.9 | 87.0 | 88.8 | 102.1 |
| 24 | 217.4 | 108.7 | 112.2 | 103.2 |

# References

1 Z. Teng, Q. Zhang, H. Yang, et al., “Atomically Dispersed Antimony on Carbon Nitride for the Artificial Photosynthesis of Hydrogen Peroxide,” *Nature Catalysis* 4, no. 5 (2021): 374-384, <https://doi.org/10.1038/s41929-021-00605-1>.

2 G. F. Abass, A. Ahmad, C. Yang, et al., “Reassessing Structural Models of Graphitic Carbon Nitride for Reliable Photocatalytic Predictions,” *Materials Advances* 7, no. 8 (2026): 4234-4247, <https://doi.org/10.1039/D6MA00065G>.

3 P. Sun, K. Zhong, X. Huang, et al., “Synergistic Utilization of Photogenerated Electrons and Holes in Carbon Nitride Nanosheet Assembly for Enhanced Photocatalytic H_2_O_2_ Production,” *Applied Catalysis B: Environment and Energy* 366, no. (2025): 124998, <https://doi.org/10.1016/j.apcatb.2024.124998>.

4 J. Yang, H. Yin, A. Du, et al., “Unveiling O_2_ Adsorption on Non-Metallic Active Site for Selective Photocatalytic H_2_O_2_ Production,” *Applied Catalysis B: Environment and Energy* 361, no. (2025): 124586, <https://doi.org/10.1016/j.apcatb.2024.124586>.

5 X. Qin, L. Zhang, M. Xu, et al., “Efficient Photocatalytic Production of H_2_O_2_ over K-Doped Carbon Nitride Via Magnetic Field-Modulated Oxygen Reduction,” *Applied Catalysis B: Environment and Energy* 362, no. (2025): 124723, <https://doi.org/10.1016/j.apcatb.2024.124723>.

6 J. Li, Y. Li, S. Wei, et al., “Asymmetric Framework Engineering of Carbon Nitride Nanotubes for Polarization Promoted H_2_O_2_ Photosynthesis,” *Applied Catalysis B: Environment and Energy* 366, no. (2025): 125073, <https://doi.org/10.1016/j.apcatb.2025.125073>.

7 P. Sun, Z. Chen, J. Zhang, et al., “Simultaneously Tuning Electronic Reaction Pathway and Photoactivity of P, O Modified Cyano-Rich Carbon Nitride Enhances the Photosynthesis of H_2_O_2_,” *Applied Catalysis B: Environmental* 342, no. (2024): 123337, <https://doi.org/10.1016/j.apcatb.2023.123337>.

8 Y. Shen, J. Shi, Y. Wang, et al., “Incorporation of Hydroxyl Groups and Π-Rich Electron Domains into g-C_3_N_4_ Framework for Boosted Sacrificial Agent-Free Photocatalytic H_2_O_2_ Production,” *Chemical Engineering Journal* 498, no. (2024): 155774, <https://doi.org/10.1016/j.cej.2024.155774>.

9 Z. Zhang, P. Luo, L. Gan, et al., “Efficient Dual-Channel Photocatalytic H_2_O_2_ Evolution and Photocatalysis-Self-Fenton Process on Defected Carbon Doped g-C_3_N_4_,” *Applied Surface Science* 649, no. (2024): 159118, <https://doi.org/10.1016/j.apsusc.2023.159118>.

10 Y. Lu, Y. Guo, S. Zhang, et al., “Promoting Proton Donation through Hydrogen Bond Breaking on Carbon Nitride for Enhanced H_2_O_2_ Photosynthesis,” *ACS Nano* 18, no. 31 (2024): 20435-20448, <https://doi.org/10.1021/acsnano.4c04797>.

11 Y. Luo, Y. Lin, Z. Weng, et al., “Rational Design of Donor-Acceptor Engineered g-C_3_N_4_ for Boosted H2O2 Production Via Photocatalytic O_2_ Reduction,” *Journal of Environmental Chemical Engineering* 11, no. 2 (2023): 109426, <https://doi.org/10.1016/j.jece.2023.109426>.

12 G. Yu, K. Gong, C. Xing, et al., “Dual P-Doped-Site Modified Porous g-C_3_N_4_ Achieves High Dissociation and Mobility Efficiency for Photocatalytic H_2_O_2_ Production,” *Chemical Engineering Journal* 461, no. (2023): 142140, <https://doi.org/10.1016/j.cej.2023.142140>.

13 W. Ren, Q. Chang, N. Li, et al., “Carbon Dots-Modulated Covalent Triazine Frameworks with Exceptionally Rapid Hydrogen Peroxide Production in Water,” *Chemical Engineering Journal* 451, no. (2023): 139035, <https://doi.org/10.1016/j.cej.2022.139035>.

14 X. Zhang, P. Ma, C. Wang, et al., “Unravelling the Dual Defect Sites in Graphite Carbon Nitride for Ultra-High Photocatalytic H_2_O_2_ Evolution,” *Energy & Environmental Science* 15, no. 2 (2022): 830-842, <https://doi.org/10.1039/D1EE02369A>.

15 J. Zhang, J. Lang, Y. Wei, et al., “Efficient Photocatalytic H_2_O_2_ Production from Oxygen and Pure Water over Graphitic Carbon Nitride Decorated by Oxidative Red Phosphorus,” *Applied Catalysis B: Environmental* 298, no. (2021): 120522, <https://doi.org/10.1016/j.apcatb.2021.120522>.

16 L. Chen, C. Chen, Z. Yang, et al., “Simultaneously Tuning Band Structure and Oxygen Reduction Pathway toward High-Efficient Photocatalytic Hydrogen Peroxide Production Using Cyano-Rich Graphitic Carbon Nitride,” *Advanced Functional Materials* 31, no. 46 (2021): 2105731, <https://doi.org/10.1002/adfm.202105731>.

17 Y. Wang, D. Meng, X. Zhao, “Visible-Light-Driven H_2_O_2_ Production from O_2_ Reduction with Nitrogen Vacancy-Rich and Porous Graphitic Carbon Nitride,” *Applied Catalysis B: Environmental* 273, no. (2020): 119064, <https://doi.org/10.1016/j.apcatb.2020.119064>.

18 S. Samanta, R. Yadav, A. Kumar, et al., “Surface Modified C, O Co-Doped Polymeric g-C_3_N_4_ as an Efficient Photocatalyst for Visible Light Assisted CO_2_ Reduction and H_2_O_2_ Production,” *Applied Catalysis B: Environmental* 259, no. (2019): 118054, <https://doi.org/10.1016/j.apcatb.2019.118054>.

19 Y. Fu, C. A. Liu, M. Zhang, et al., “Photocatalytic H_2_O_2_ and H_2_ Generation from Living Chlorella Vulgaris and Carbon Micro Particle Comodified g-C3N4,” *Advanced Energy Materials* 8, no. 34 (2018): 1802525, <https://doi.org/10.1002/aenm.201802525>.

20 Z. Wei, M. Liu, Z. Zhang, et al., “Efficient Visible-Light-Driven Selective Oxygen Reduction to Hydrogen Peroxide by Oxygen-Enriched Graphitic Carbon Nitride Polymers,” *Energy & Environmental Science* 11, no. 9 (2018): 2581-2589, <https://doi.org/10.1039/C8EE01316K>.

21 Z. Zhu, H. Pan, M. Murugananthan, et al., “Visible Light-Driven Photocatalytically Active g-C_3_N_4_ Material for Enhanced Generation of H_2_O_2_,” *Applied Catalysis B: Environmental* 232, no. (2018): 19-25, <https://doi.org/10.1016/j.apcatb.2018.03.035>.

22 Y. Kofuji, Y. Isobe, Y. Shiraishi, et al., “Carbon Nitride–Aromatic Diimide–Graphene Nanohybrids: Metal-Free Photocatalysts for Solar-to-Hydrogen Peroxide Energy Conversion with 0.2% Efficiency,” *Journal of the American Chemical Society* 138, no. 31 (2016): 10019-10025, <https://doi.org/10.1021/jacs.6b05806>.

23 L. Yang, G. Dong, D. L. Jacobs, et al., “Two-Channel Photocatalytic Production of H_2_O_2_ over g-C_3_N_4_ Nanosheets Modified with Perylene Imides,” *Journal of Catalysis* 352, no. (2017): 274-281, <https://doi.org/10.1016/j.jcat.2017.05.010>.

24 Y. Shiraishi, S. Kanazawa, Y. Kofuji, et al., “Sunlight-Driven Hydrogen Peroxide Production from Water and Molecular Oxygen by Metal-Free Photocatalysts,” *Angewandte Chemie International Edition* 53, no. 49 (2014): 13454-13459, <https://doi.org/10.1002/anie.201407938>.
